# Metabolite profiling of the carnivorous pitcher plants *Darlingtonia* and *Sarracenia*

**DOI:** 10.1371/journal.pone.0171078

**Published:** 2017-02-21

**Authors:** Hannu Hotti, Peddinti Gopalacharyulu, Tuulikki Seppänen-Laakso, Heiko Rischer

**Affiliations:** 1 VTT Technical Research Centre of Finland Ltd, Espoo, Finland; 2 Institute for Molecular Medicine Finland (FIMM), University of Helsinki, Helsinki, Finland; Tallinn University of Technology, ESTONIA

## Abstract

Sarraceniaceae is a New World carnivorous plant family comprising three genera: *Darlingtonia*, *Heliamphora*, and *Sarracenia*. The plants occur in nutrient-poor environments and have developed insectivorous capability in order to supplement their nutrient uptake. *Sarracenia flava* contains the alkaloid coniine, otherwise only found in *Conium maculatum*, in which its biosynthesis has been studied, and several *Aloe* species. Its ecological role and biosynthetic origin in *S*. *flava* is speculative. The aim of the current research was to investigate the occurrence of coniine in *Sarracenia* and *Darlingtonia* and to identify common constituents of both genera, unique compounds for individual variants and floral scent chemicals. In this comprehensive metabolic profiling study, we looked for compound patterns that are associated with the taxonomy of *Sarracenia* species. In total, 57 different *Sarracenia* and *D*. *californica* accessions were used for metabolite content screening by gas chromatography-mass spectrometry. The resulting high-dimensional data were studied using a data mining approach. The two genera are characterized by a large number of metabolites and huge chemical diversity between different species. By applying feature selection for clustering and by integrating new biochemical data with existing phylogenetic data, we were able to demonstrate that the chemical composition of the species can be explained by their known classification. Although transcriptome analysis did not reveal a candidate gene for coniine biosynthesis, the use of a sensitive selected ion monitoring method enabled the detection of coniine in eight *Sarracenia* species, showing that it is more widespread in this genus than previously believed.

## Introduction

Sarraceniaceae is a New World carnivorous plant family comprising three genera: *Darlingtonia* Torr. (monotypic), *Heliamphora* Benth. (ca. 23 species [1]) and *Sarracenia* L. (ca. 11 species [2]). The distribution of *Darlingtonia* is limited to a few locations along the western coast of North America, *Heliamphora* occurs mainly on tepuis of the Guiana Highlands in South America and *Sarracenia* is the most widespread genus in the family, found in the eastern coastal plains of North America. *Darlingtonia californica*, *Sarracenia*, and *Heliamphora* are able to compete in nutrient-poor habitats due to their insectivorous nature, *i*.*e*. the ability to attract, capture, and digest insects to supplement their nutrient uptake. A common feature for all three genera is that they lure insects to their elongated tubular leaves. In order to attract insects, they produce extrafloral nectar [3], emit insect attractants [4], and most species are brightly colored. They utilize various methods to capture their prey. *Darlingtonia californica* and *S*. *psittacina*, for example, hide their entry/exit hole from the inside, displaying multiple translucent false exits so that insects finally get exhausted and fall into the pitcher. Other *Sarracenia* and *Heliamphora* species utilize downward pointing hairs and waxy surfaces in their pitchers in order to trap insects.

The family is relatively poorly described in terms of chemical constituents [5], which is surprising given the fact that *Sarracenia* species have long been used as traditional medicine by many aboriginal communities in North America, and have attracted renewed pharmaceutical interest due to recent investigations revealing their cytoprotective activities in cell models [6]. *Darlingtonia californica* has not been chemically investigated at all to date, but several insect-attracting constituents have been described from the spoon-shaped lid structures of pitchers of two *Heliamphora* species [4]. Various compounds found in *Sarracenia* have also been reported, including volatiles [7,8], flavonoids [9–11], phytochemicals [12–14] and pitcher fluid composition [3,11,15,16]. Sarracenin, an enol diacetal monoterpene, was first identified in *S*. *flava* [17] and later found in a number of *Sarracenia* [18] and *Heliamphora* [4] species. *Sarracenia flava* is the most studied species with respect to its chemical composition [7,8,19,20]. Interestingly, *S*. *flava* contains coniine [21], a toxic alkaloid, which is otherwise only known from the unrelated *Conium maculatum* (Apiaceae) and several *Aloe* species (Xanthorrhoeaceae) [22,23]. In *C*. *maculatum*, a polyketide synthase (PKS) initiates the biosynthesis of coniine [24]. The original study [21] referred to earlier research on *S*. *purpurea*, indicating that it could also contain coniine or related alkaloids. Mody et al. [21] speculated that coniine in *S*. *flava* paralyzes insects, whereas Harborne [25] postulated insect attraction. Systematic investigations of the compound’s wider occurrence in the genus have hitherto not been performed. In order to follow up on the earlier findings in *S*. *flava* and to expand our knowledge on coniine distribution in *Sarracenia*, we aimed at investigating a number of accessions using a sensitive gas chromatography-mass spectrometry (GC-MS) method applying selected ion monitoring (SIM) to detect coniine reliably in plant material even at low concentrations. Additionally, the transcriptomes of *S*. *psittacina* and *S*. *purpurea* were analysed for encoded candidate PKSs putatively involved in coniine biosynthesis.

Three previous studies derived the phylogeny of *Sarraceniaceae* using gene sequence data, with incongruent results [26–28]. Stephens et al. [2] recently addressed this inconsistency by applying a target enrichment approach to assess the phylogenetic relationships among 75 *Sarracenia* accessions. Unlike the mutations from highly conserved genomic loci, the chemical composition usually differs even between closely related species and hence is not suitable for deriving reliable taxonomies [29]. In a biochemical profiling study of volatiles, Jürgens et al. [8] applied an approach based on multidimensional scaling to study the similarities among different species. They then used similarity percentage analysis (SIMPER) to obtain compounds that explained the highest amount of dissimilarity among the samples. Thus, Jürgens et al. [8] focused on the variability in the data without focusing directly on the phylogenetic structure. The phylogeny, on the other hand, may explain the chemical diversity of the species. The aim of our current study was to provide a comprehensive catalogue of chemical constituents of *Sarraceniaceae* and to examine the extent to which the known phylogenetic information explains the chemical composition of the plants. Therefore, we employed a comprehensive metabolic profiling approach using GC-MS to detect all ions in SCAN mode in a large sample collection. The genus *Sarracenia* comprises 44 recognised intraspecific taxa [30] within 11 *Sarracenia* species [2]. By contrast, only one *Darlingtonia* species is known, occurring in a geographically restricted area. Our collection was selected to cover adequately the diversity of pitcher plants that had been examined in phylogenetic studies [26]. We found common chemical constituents among the plants, unique compounds for individual variants and possible floral scent chemicals as classified according to Knudsen et al. [29], and studied whether the biochemical profiles can be explained by the taxonomy presented in Stephens et al. [2].

## Materials and methods

### Plant material

Pitchers of cultivated plants were investigated in order to exclude environmental effects. *Sarracenia* L. (56 accessions) and *Darlingtonia californica* Torr. were provided by C. Klein, Germany (http://www.carnivorsandmore.de). Metabolite and coniine content screening was performed using global metabolomics in a set of 48 accessions (Table 1) that contained one *D*. *californica* and 47 *Sarracenia* accessions. The *Sarracenia* accessions included *S*. *alata* Alph.Wood (5 accessions), *S*. *flava* L. (11 accessions), *S*. *leucophylla* Raf. (5 accessions), *S*. *minor* Walt. (3 accessions), *S*. *oreophila* (Kearney) Wherry (2 accessions), *S*. *psittacina* Michx. (4 accessions), *S*. *purpurea* L. (13 accessions) and *S*. *rubra* Walt. (4 accessions).

**Table 1:** List of *Darlingtonia* and *Sarracenia* accessions for metabolite profiling by GC-MS (SCAN).

Species	Newer classification (according to [2])	Sample number	Sample numbering in [2]^a^	Growth form	Origin	Coniine in Lid			Coniine in Pitcher		
						*m/z* 80	*m/z* 84	*m/z* 126	*m/z* 80	*m/z* 84	*m/z* 126
*Darlingtonia californica*		18	SAMN03354579			-	x^f^	-	-	x	-
*Sarracenia alata*		14	SAMN03354583^b^	blood form	DeSoto, Mississippi	x^g^	x^g^^,^^h^	x^h^	-	x	-
*Sarracenia alata*		46	SAMN03354583^b^	blood form	Stone, Mississippi	x^i^	x^f^^,^^i^	-	x^i^	x^f^^,^^i^	-
*Sarracenia alata*		28	SAMN03354584^c^		Citronelle, Alabama	x	x^h^	-	x	x^h^	-
*Sarracenia alata*		40	SAMN03354586^b^		Robertson, Texas	-	x	-	x^i^	x^i^	-
*Sarracenia alata*		42	SAMN03354583^b^		Perry Co. Mississippi	-	x	-	x^i^	x^i^	-
*Sarracenia flava*		20	SAMN03354588^d^			x	-	-	x	x^h^	-
*Sarracenia flava* var. *atropurpurea*		31	SAMN03354589^b^		Bloodwater, Florida	x^i^	x^i^	x	x^i^	x^i^	x
*Sarracenia flava* var. *atropurpurea*		35	SAMN03354589^b^		Bay County, Florida	x	x^h^	-	x	x^h^	-
*Sarracenia flava* var. *atropurpurea*		1	SAMN03354589^b^		Bloodwater, Florida	x	x^h^	-	-	x^f^	-
*Sarracenia flava* var. *cuprea*		10	SAMN03354591^d^			x	x^f^	-	x	x^h^	-
*Sarracenia flava* var. *flava*		11	SAMN03354593^b^		Dinwiddie, Virginia	x^g^	x^g^^,^^h^	x^g^	x^g^	x^g^^,^^h^	x^g^
*Sarracenia flava* var. *heterophylla*		21	SAMN03354590^b^		near Shallotte, North Carolina	x^i^	x^i^	-	x^g^	x^g^^,^^h^	x^g^
*Sarracenia flava* var. *maxima*		44	SAMN03354593^d^			x	x^h^	-	x^g^	x^g^^,^^h^	x^g^
*Sarracenia flava* var. *ornata*		29	SAMN03354592^b^		Sandy Creek, North Carolina	x	x^h^	x^j^	x^g^	x^g^^,^^h^	x^g^
*Sarracenia flava* var. *rubricorpora*		8	SAMN03354594^b^		Apalachicola, Florida	x^g^	x^g^^,^^h^	x^g^	x^i^	x^i^	-
*Sarracenia flava* var. *rugelii*		32	SAMN03354596^d^			x^i^	x^i^	-	x^i^	x^f^^,^^i^	-
*Sarracenia leucophylla*		33	SAMN03354604^b^		Splinter Hills Bog, Alabama	-	x	-	x	x^h^	-
*Sarracenia leucophylla*		17	SAMN03354603^d^	Big pink lip	Apalachicola, Florida	x^g^	x^g^^,^^h^	x^g^	x^i^	x^i^	-
*Sarracenia leucophylla*		12	SAMN03354605^d^	Pubescent, covered with white hairs		-	x	x	x	x^h^	-
*Sarracenia leucophylla* 'Schnell's Ghost'		45	SAMN03354606^d^			-	x	x	x^i^	x^f^^,^^i^	-
*Sarracenia leucophylla* var. *alba*		26	SAMN03354608^d^			x^g^	x^g^^,^^h^	x^g^	x^g^	x^g^^,^^h^	x^g^
*Sarracenia minor*		15	SAMN03354609^d^	large form		x	x^f^	-	x^i^	x^i^	-
*Sarracenia minor*		4	SAMN03354610^d^	small form		-	x	-	x^i^	x^i^	-
*Sarracenia minor* var. *okefenokeensis*		5	SAMN03354614^e^			x	x^h^	-	x	x^h^	-
*Sarracenia oreophila*		22	SAMN03354616^d^			x^g^	x^g^^,^^h^	x^g^	x^g^	x^g^^,^^h^	x^g^
*Sarracenia oreophila*		27	SAMN03354615^d^		Sand Hill, Alabama	x^g^	x^g^^,^^h^	x^g^	x	x^h^	-
*Sarracenia psittacina* f. *heterophylla*		6	SAMN03354621^d^	Yellow flower		x^g^	x^g^^,^^h^	x^g^	x	x^h^	-
*Sarracenia psittacina* f. *heterophylla*		24	SAMN03354623^b^^,^^d^		Baldwin County, Alabama	x	x^h^	-	x^i^	x^i^	-
*Sarracenia psittacina*		13	SAMN03354626^b^	Gulf giant	Wewahitchka, Florida	-	X	-	x^g^	x^g^^,^^h^	x^g^
*Sarracenia psittacina*		43	SAMN03354628^e^	Yellow flower		x	x^h^	-	x	x^h^	-
*Sarracenia purpurea* subsp. *purpurea*		16	SAMN03354629^e^		Switzerland	x	x^f^	-	x	x^h^	-
*Sarracenia purpurea* subsp. *purpurea*		19	SAMN03354630^e^			x^g^	x^g^^,^^h^	x^g^	x^g^	x^g^^,^^h^	x^g^
*Sarracenia purpurea* subsp. *purpurea* f. *heterophylla*		38	SAMN03354631^e^	extreme dense growth form		-	x	-	x	x^h^	-
*Sarracenia purpurea* subsp. *venosa*		36	SAMN03354633^d^^,^^e^			-	x	-	x	x^i^	-
*Sarracenia purpurea* subsp. *venosa*		47	SAMN03354634^d^^,^^e^		Tom's Swamp	-	x	-	-	x	-
*Sarracenia purpurea* subsp. *venosa*		30	SAMN03354663^d^^,^^e^	All green		x	x^i^	-	-	x	-
*Sarracenia purpurea* subsp. *venosa*		37	SAMN03354632^b^		Tyrrel County, North Carolina	-	x	-	-	x^f^	-
*Sarracenia purpurea* subsp. *venosa* var. *burkii*	S. rosea	34	SAMN03354637^d^^,^^e^	small strongly waving form		x^i^	x^f^^,^^i^	-	x	x^h^	-
*Sarracenia purpurea* subsp. *venosa* var. *burkii*	S. rosea	7	SAMN03354640^d^^,^^e^		Carteret, North Carolina	x	x^h^	-	x	x^h^	-
*Sarracenia purpurea* subsp. *venosa* var. *burkii*	S. rosea	39	SAMN03354639^d^^,^^e^	Giant		x^i^	x^f^^,^^i^	-	x	x^h^	-
*Sarracenia purpurea* subsp. *venosa* var. *burkii* f. *luteola*	S. rosea f. luteola	48	SAMN03354638^d^^,^^e^	veinless form		x	x^h^	-	x^g^	x^g^^,^^h^	x^g^
*Sarracenia purpurea* subsp. *venosa* var. *montana*		41	SAMN03354635^e^			x^g^	x^g^^,^^h^	x^g^	†	†	†
*Sarracenia purpurea* subsp. *venosa* var. *montana*		9	SAMN03354636^e^		Chipola, Florida	x^g^	x^g^^,^^g^	x^g^	x^i^	x^i^	-
*Sarracenia rubra* subsp. *alabamensis*	S. alabamensis	2	SAMN03354582^e^			x	x^h^	-	x	x^h^	-
*Sarracenia rubra* subsp. *gulfensis*		25	SAMN03354647^d^			x	x^h^	-	-	x^f^	-
*Sarracenia rubra* subsp. *jonesii*	S. jonesii	3	SAMN03354599^b^		Cesars Head, South Carolina	-	-	-	-	-	-
*Sarracenia rubra* subsp. *wherryi*	S. alabamensis subsp. wherryi	23	SAMN03354650^e^			x	x^h^	-	x	x^h^	-

x mass *(m/z)* present,—not present

† not analysed.

^a^ Given a corresponding sample when applicable, otherwise c.

^b^ Based on collection location.

^c^ Mississippi accessions were used as they are the closest geographical location for this sample.

^d^ Drawn lots, if there were more than two options from which to choose.

^e^ Based on the same variety if collection location is not available.

^f^ Low intensity fragment.

^g^ Masses *m/z* 80, 84 and 126 are present in correct proportions.

^h^ Mass *m/z* 80 has greater intensity than *m/z* 84.

^i^ Masses (*m/z*) have the same relative intensity.

^j^ Mass *m/z* 126 has the greatest intensity of the three selected ions.

Targeted metabolomics for sensitive detection of coniine was performed in 17 accessions, including eight accessions that were also analyzed using global metabolomics (Table 2). These accessions included *S*. *alata* (2 accessions), *S*. *flava* (4 accessions), *S*. *leucophylla* (1 accession), *S*. *minor* (1 accession), *S*. *oreophila* (1 accession), *S*. *psittacina* (2 accessions), *S*. *purpurea* (4 accessions) and *S*. *rubra* (2 accessions).

**Table 2:** *Sarracenia* accessions for targeted coniine analysis by GC-MS (SIM).

Species	Newer classification (according to [2])	Sample number^1^	Growth form	Origin	Coniine in Lid	Coniine in Pitcher
*Sarracenia* alata 'Black Tube'					x	x*
*Sarracenia alata*			Wide hood	Stane County, Mississippi	x	x
*Sarracenia flava*		20			x*	-
*Sarracenia flava* var. *atropurpurea*					x*	x*
*Sarracenia flava* var. *maxima*		44			x*	x*
*Sarracenia flava* var. *ornata*					x	x*
*Sarracenia leucophylla*				Citronelle, Alabama	x	†
*Sarracenia minor* var. *okefenokeensis*		5			x	-
*Sarracenia oreophila*			typical form		-	x
*Sarracenia psittacina*		13	Gulf giant		†	x
*Sarracenia psittacina*		43	Yellow flower		x	x
*Sarracenia purpurea* subsp. *burkii*			Veinless		x	x
*Sarracenia purpurea* subsp. *venosa*		36			x	x
*Sarracenia purpurea* subsp. *venosa* var. *burkii* f. *luteola*	S. rosea f. luteola	48	veinless form		x	x
*Sarracenia purpurea* subsp. *venosa* var. *montana*		41			x	x
*Sarracenia rubra* subsp. *alabamensis*	S. alabamensis			Chilton County, Alabama	x	x
*Sarracenia rubra* subsp. *gulfensis*					x	x

x present;—not present; x* trace, close to limit of detection (1 μg/ml)

† not analysed.

^1^ Included in metabolite profiling (Table 1).

Cultivated poison hemlock (*Conium maculatum* L.) and barley (*Hordeum vulgare* L. ‘Golden Promise’), were used as alkaloid-containing or alkaloid-free reference material, respectively.

### Metabolite extraction

Lids and pitchers were separated, washed with tap water and ground up. Fresh (2 g; metabolite profiling) or freeze dried (200 mg; coniine analysis) plant material was used for extraction as described in [31]. Lipids were removed from the plant material with 3.0 ml petroleum ether (puriss p.a., Sigma-Aldrich Munich, Germany). The plant material was diluted with 2.0 ml ultrapure water and a pH above 9 was obtained by addition of 10% ammonium hydroxide solution (25% stock solution, pro analysi, Merck KGaA, Darmstadt, Germany). Metabolites were extracted twice with 2.0 ml dichloromethane (HPLC grade, Rathburn Chemicals Ltd, Walkerburn, Scotland, UK). The combined dichloromethane extracts were evaporated to dryness and dissolved in 100 μl dichloromethane for further analysis.

### Gas chromatography-mass spectrometry

Samples (1 μl) were analysed by GC-MS consisting of a 6890A Series GC (Agilent Technologies, Inc., Santa Clara, CA, USA) combined with an Agilent 5973 Network MSD and a Combipal automatic sampler (Varian Inc., Palo Alto, CA, USA). Analytes were separated by an Agilent HP-5MS capillary column (25 m × 0.2 mm i.d, 0.33 μm). The temperature program started at 50°C with 1 min holding time and then increased at 10°C/min up to 300°C. MSD was operated in electron impact mode at 70 eV.

Pure coniine (Sigma-Aldrich, Munich, Germany) was used as the reference compound in developing the GC-MS method. To determine the detection limit of coniine in the SIM-method, 1, 5, 10 and 20 μg was spiked into alkaline water and extracted as described in [31]. Cotinine (20 μg/sample) (Sigma-Aldrich, Munich, Germany) was used as an internal standard.

### PKS-encoding genes in transcriptomes of *S*. *psittacina* and *S*. *purpurea*

Available transcriptomes of *S*. *psittacina* (accession number SRX060168 in the NCBI database) and *S*. *purpurea* (accession number SRX060177 in the NCBI database) [32] were analyzed for PKSs using Geneious (version 9.0.4) [33]. The tblastn algorithm in Geneious was used to search the sequence database with the *Medicago sativa* CHS2 amino acid sequence [34] as the template and a stringency setting of 1e-10. The obtained nucleotide sequence hits were translated to amino acid sequences, and the correct reading frames were chosen and aligned using the Geneious alignment option.

### Data handling

Peaks in GC-MS chromatograms were integrated automatically using MSD ChemStation software (version E.02.01.1177, Agilent Technologies, Inc., Santa Clara, CA, USA). Peaks were identified with the Palisade Complete 600K Mass Spectral Library (Palisade Mass Spectrometry, Ithaca, NY, USA) and the NIST Mass Spectral Search Program (The Standard Reference Data Program of the National Institute of Standards and Technology, Gaithersburg, MD, USA). The computer-generated identifications were sorted manually, with a cut-off at 70% identification [35], into an Excel spreadsheet (Microsoft, Redmond, WA, USA) according to their chemical structure, elution time and origin. When peaks with same retention time were identified as different hydrocarbons in multiple samples, they were treated as n-alkanes at the specific retention time. The relative peak abundances were used in the data input.

### Data mining

The metabolite data were treated in two formats: (1) a qualitative format representing presence (*i*.*e*. concentration level above the detection limit) or absence (concentration level below the detection limit) of a compound in a sample, by coding the presence and absence as 1 and 0, respectively, and (2) a quantitative or continuous format in which the concentration level is given as the percentage of the total peak area. The main aim of our data mining was to visualize any patterns present in the data. Towards this goal, it was first noted that the current data are very high dimensional (*i*.*e*. contain a large number of compounds), very sparse (91.35% zeros in the lids dataset and 91.86% in the pitchers dataset), and that the distinct species show huge chemical diversity (*i*.*e*. the metabolite composition of different plants is largely distinct). Therefore, it is reasonable to expect that only a small proportion of compounds are likely to be useful for clustering the samples. A feature selection approach for clustering [36] was applied in order to identify the most important features required for deriving hierarchical clusters. This approach computes and reweights the overall dissimilarity matrix while applying a lasso-type penalty, which results in a dissimilarity matrix sparse in features [36]. This sparse clustering was applied using the R package sparcl. In order to compute the hierarchical clustering with the qualitative format of the data, the hamming distance was used as the dissimilarity measure. For the quantitative format of the data, the Euclidean distance was used. The complete linkage method was used for the clustering.

**Fig 1:**
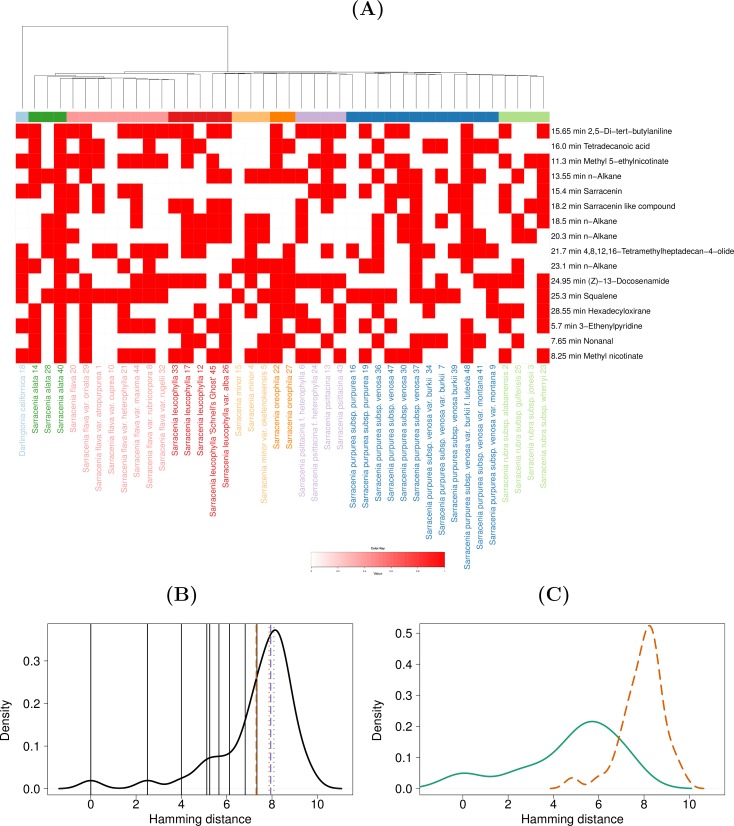
Visualization of selected metabolite features from the qualitative data of lids. (A) Heat map visualization of selected metabolite features from the qualitative data of lids. The phylogenetic tree from [2] is displayed as the column dendrogram. Six samples of our dataset (11, 31, 35, 38, 42, and 46) are omitted from this heat map based on the sample selection procedure described in the Methods section. (B) Comparison of average within-clade distances (aWCDs) against the background distribution of average species-level distances (aSLDs) and average between-clade distances (aBCDs). Distribution of aSLDs was calculated using qualitative data of the selected metabolite features and displayed in a density plot. The black vertical lines mark the individual aWCDs. The orange dashed and dotted lines show the mean and median of aSLDs. The purple dashed and dotted lines show the mean and median of aBCDs. (C) Comparison of aWCDs (green continuous density line) with aBCDs (orange dashed density line).

**Fig 2:**
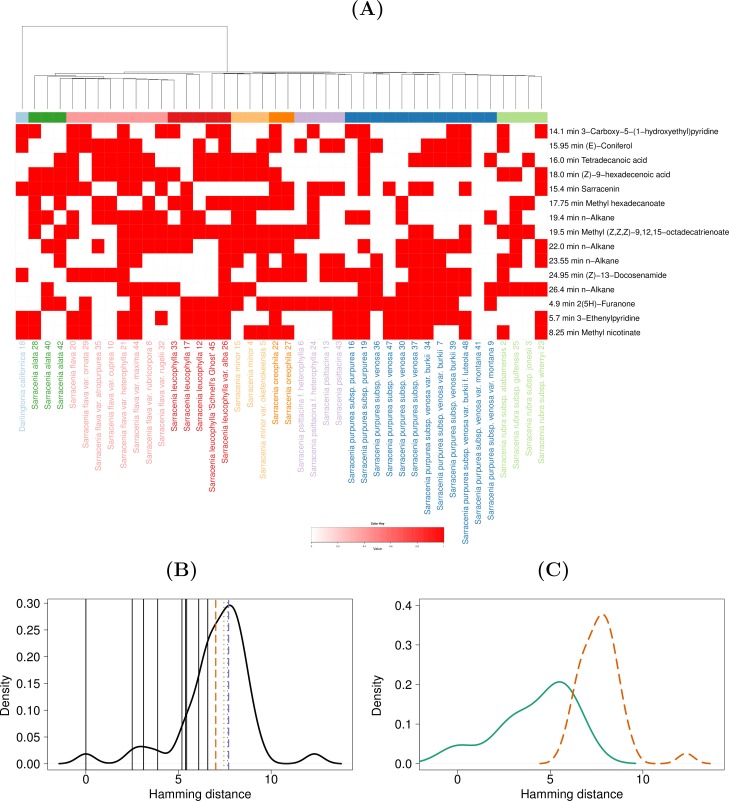
Visualization of selected metabolite features from the qualitative data of pitchers. (A) Heat map visualization of selected metabolite features from the qualitative data of pitchers. The phylogenetic tree from [2] is displayed as the column dendrogram. Six samples of our dataset (1, 11, 14, 31, 38, and 46) are omitted from this heat map, based on the sample selection procedure described in the Methods section. (B) Comparison of average within-clade distances (aWCDs) against the background distribution of average species-level distances (aSLDs) and average between-clade distances (aBCDs). Distribution of aSLDs was calculated using qualitative data of the selected metabolite features and displayed in a density plot. The black vertical lines mark the individual aWCDs. The orange dashed and dotted lines show the mean and median of aSLDs. The purple dashed and dotted lines show the mean and median of aBCDs. (C) Comparison of aWCDs (green continuous density line) with aBCDs (orange dashed density line).

In order to compare the phylogenetic structure with the chemical profiles, the MP-EST accession tree from [2] was downloaded. Then the accessions in the two studies were mapped based on the location of sample collection, which resulted in a many-to-many mapping (Table 1) with one or more of 42 nodes in the phylogenetic tree matching one or more of 48 species in our study. From this, 36 possible bijective maps were enumerated, and compound-based distances corresponding to each bijective map were calculated as follows. The distance between every pair of accessions was calculated using hamming distance for the binary and Euclidean distance for the continuous data of the selected metabolite features. These distances are referred to below as species-level distances (SLD). Using the clades resolved in the MP-EST accession tree (*i*.*e*. *D*. *californica*, *S*. *flava*, *S*. *psittacina*, *S*. *minor*, *S*. *purpurea* complex, *S*. *rubra* complex, *S*. *alata*, *S*. *leucophylla*, and *S*. *oreophila*), distances within and between the clades were calculated. A within-clade distance (WCD) was calculated as the average of all pairwise SLDs of accessions within the clade. A between-clade distance (BCD) was calculated as the average of all SLDs of accession-pairs across the pair of clades. Average species-level and clade-level distance matrices were calculated over all 36 bijective maps to derive the average within-clade (aWCD) and between-clade distances (aBCD), as well as the average species-level distances (aSLD). These averaged distances were used to assess how well the metabolite data supports the phylogenetic structure. If the phylogenetic structure explains the compound data, the aWCDs are expected to be lower than the aBCDs. This was assessed by comparing aWCDs against not only aBCDs but also aSLDs as an additional test. More precisely, we (I) visualized aWCDs against the background distance distribution formed by aSLDs (Fig 1B and Fig 2B, S1B and S2B Figs), (II) visualized the difference between the distribution of aWCDs and aBCDs (Fig 1C and Fig 2C, S1C and S2C Figs) and (III) performed one-sided Wilcoxon’s rank sum tests to assess whether aWCDs are less prevalent than aBCDs.

In order to visualize the metabolite features selected for clustering alongside the phylogenetic structure presented in [2], the best mapping of samples between the MP-EST accession tree and our compound data was obtained. The best bijective map is expected to result in the maximum BCD and minimum WCD among all possible bijective maps. To achieve this objective, we chose the map that yields the maximum difference between the mean values of BCD and WCD *i*.*e*. *mean*(BCD)*–mean*(WCD) for these visualizations (Fig 1A and Fig 2A, S1A and S2A Figs). Thus, the heat maps shown in Fig 1A and Fig 2A, S1A and S2A Figs contain only one sample from our compound dataset for each node in the MP-EST accession tree chosen to maximize the *mean*(BCD)*–mean*(WCD). Since only 42 nodes in the accession tree map to our dataset, each heat map omits 6 samples from our study. In particular, the samples numbered 31 and 46 (Table 1) were omitted in all four heat maps (Fig 1A and Fig 2A, S1A and S2A Figs). Apart from these two samples, 11, 35, 38, and 42 were omitted from Fig 2A; 14, 35, 37, and 44 were omitted from S1A Fig; 1, 11, 14, and 38 were omitted from Fig 2A; and 1, 11, 38, and 42 were omitted from S2A Fig.

All the statistical analyses and visualizations were performed using the R statistical software [37] and its packages such as gplots, sparcl, metadar (http://code.google.com/p/metadar), ihm (http://code.google.com/p/ihm), and RColorBrewer.

## Results

### Coniine identification and occurrence in *Sarracenia*

**Fig 3:**
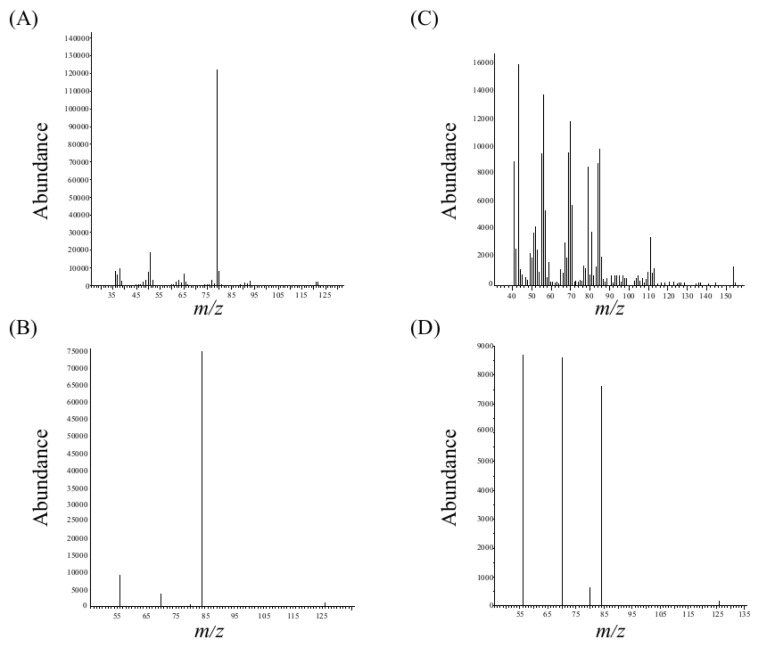
Mass spectrum of coniine reference substance and detection of coniine in the sample matrix. Mass spectrum of pure coniine in SCAN mode (A) and selected fragments in SIM mode (B). Coniine detection in sample matrix (*S*. *flava)* in SCAN (C) and SIM modes (D).

With the GC-MS method used, coniine elutes at a constant retention time (6.33±0.01min) even in spiked barley material and *C*. *maculatum* leaf extract. The samples were analysed on the basis of their SCAN mass spectra and were compared to a database. Pure coniine matched the database with 86%, or in plant matrix with 78%-86% identity. The retention time of coniine was very stable, and the ions 80, 84, and 126 exhibited the same relative abundances in the sample matrix and in the coniine reference substance (Fig 3). Therefore, a match lower than 90% can be considered acceptable. Using the SCAN mode, coniine was detected in *S*. *alata*, *S*. *flava*, *S*. *leucophylla*, *S*. *oreophila*, *S*. *psittacina* and *S*. *purpurea* (incl. *S*. *rosea*) (Table 1). In *D*. *californica*, only the fragment *m/z* 84 was detected, whereas in *S*. *jonesii* (3) none of the ions were detected at 6.33 min.

In order to detect coniine at low concentrations, we operated the GC-MS in SIM mode. Based on the fragmentation pattern of coniine (*m/z* 43, 56, 70, 80, 84, 97, 110, and 126), the characteristic ions *m/z* 56, 70, 80, 84 (base peak) and 126 (mass peak) were selected. The fragments *m/z* 80, 84, and 126 are specific for coniine, in contrast to the ions *m/z* 56 and 70, which are shared with many other molecules.

The limit of detection for coniine in SIM was 1 μg/ml, which corresponds to 1 μg/g dry weight. Using SIM detection, coniine was identified from *S*. *alata*, *S*. *flava*, *S*. *leucophylla*, *S*. *minor*, *S*. *oreophila*, *S*. *psittacina*, *S*. *purpurea* (incl. *S*. *rosea*) and *S*. *rubra* (incl. *S*. *alabamensis*) (Table 2). Of these, *S*. *flava* and *S*. *alata* samples only contained coniine traces close to the detection limit, whereas other samples accumulated clearly higher levels of coniine. No coniine was detected in the pitchers of *S*. *minor* var. *okefenokeensis* or the lids of *S*. *oreophila*.

### PKSs in *Sarracenia* transcriptomes

*Sarracenia psittacina* and *S*. *purpurea* transcriptomes were analysed using the tblastn algorithm with the stringency set to 1e-10 and *M*. *sativa* CHS2 as a template, resulting in 8 and 12 sequences, respectively. Correct reading frames were selected and aligned with each other after the nucleotide sequences were translated to amino acid sequences. This resulted in three unique contigs per species. Of these, one represents the N-terminus and two the C-terminus when compared to full-length PKS-enzyme. None of the contigs cover the middle part of the PKS-enzyme sequence, but they do contain all the conserved amino acids in the active site in the observed area [38] when compared to other full-length PKSs (S3 Fig).

### Metabolite profiles

The metabolite profiles of lids and pitchers were analysed separately. In addition to analysing the metabolite profiles using the quantitative (concentration) data, we also investigated the qualitative (presence or absence) data in which compounds with non-zero concentration levels (*i*.*e*. with levels above the detection limits) were treated as present and compounds with levels below the detection limits as absent.

The manually aligned lid dataset consisted of a total of 560 compounds detected in at least one sample. Among these, there were library matches (≥70%) for 69 alcohols, 70 aldehydes and ketones, 53 esters, 58 ethers, 30 carboxylic acids and sterols, 45 hydrocarbons (including some identified as alkanes), 148 n-alkanes, 75 nitrogen compounds, and 12 sulphur compounds. However, each individual plant's lid contained an average of only 48 compounds. The lid sample of *S*. *purpurea* subsp. *purpurea* (16) contained the lowest number of compounds (n = 20) and *S*. *rubra* subsp. *wherryi* (23) had the highest number of compounds (n = 85). The barplot in S4 Fig shows the distribution of compounds across all the lid samples. Furthermore, every lid sample had on average approximately six compounds uniquely found in that sample but in no other sample, one of which could be classified as a floral scent component which had previously been detected from intact flowers [29]. *Sarracenia leucophylla* (17) displayed the highest number (n = 4) of floral scent compounds (Table 3). The sample *S*. *purpurea* subsp. *venosa* var. *burkii* (39) is an exception in that it did not accumulate unique compounds, whereas *S*. *flava* var. *atropurpurea* (35) had the largest number (n = 18) of unique compounds. S1A Table shows the compounds unique to each sample along with their concentration levels. Finally, when we compared the lid samples in pairs, we observed that, on average, every lid sample contained 32 unique compounds (S2A Table).

**Table 3:** Unique compounds for each *Darlingtonia* and *Sarracenia* accession in lids and pitchers.

	Lids		Pitchers	
Species/strain	Unique compounds	Floral scent compounds [29]	Unique compounds	Floral scent compounds [29]
*Darlingtonia californica* 18	5	0	16	0
*Sarracenia alata* 14	4	1	3	1
*Sarracenia alata* 46	14	2	1	1
*Sarracenia alata* 28	3	1	2	0
*Sarracenia alata* 40	5	1	3	0
*Sarracenia alata* 42	2	0	11	0
*Sarracenia flava* 20	4	0	13	1
*Sarracenia flava* var. *ornata* 29	12	3	4	1
*Sarracenia flava* var. *atropurpurea* 31	4	0	25	6
*Sarracenia flava* var. *atropurpurea* 35	18	1	11	1
*Sarracenia flava* var. *atropurpurea* 1	10	2	11	2
*Sarracenia flava* var. *cuprea* 10	2	1	5	0
*Sarracenia flava* var. *flava* 11	9	1	12	1
*Sarracenia flava* var. *heterophylla* 21	5	2	4	1
*Sarracenia flava* var. *maxima* 44	1	0	8	1
*Sarracenia flava* var. *rubricorpora* 8	2	1	3	0
*Sarracenia flava* var. *rugelii* 32	3	0	4	0
*Sarracenia leucophylla* 33	5	0	11	0
*Sarracenia leucophylla* 17	15	4	5	1
*Sarracenia leucophylla* 12	14	3	0	0
*Sarracenia leucophylla 'Schnell's Ghost'* 45	16	3	1	0
*Sarracenia leucophylla* var. *alba* 26	10	0	19	3
*Sarracenia minor* 15	7	0	1	0
*Sarracenia minor* 4	5	2	9	0
*Sarracenia minor* var. *okefenokeensis* 5	15	3	16	6
*Sarracenia oreophila* 22	7	2	7	0
*Sarracenia oreophila* 27	6	0	3	0
*Sarracenia psittacina* f. *heterophylla* 6	1	1	0	0
*Sarracenia psittacina* f. *heterophylla* 24	3	1	1	0
*Sarracenia psittacina* 13	10	3	9	5
*Sarracenia psittacina* 43	3	0	5	0
*Sarracenia purpurea* subsp. *purpurea* 16	1	0	0	0
*Sarracenia purpurea* subsp. *purpurea* 19	1	0	8	4
*Sarracenia purpurea* subsp. *purpurea* f. *heterophylla* 38	4	1	5	2
*Sarracenia purpurea* subsp. *venosa* 36	15	2	17	1
*Sarracenia purpurea* subsp. *venosa* 47	3	0	2	0
*Sarracenia purpurea* subsp. *venosa* 30	2	0	1	0
*Sarracenia purpurea* subsp. *venosa* 37	4	2	9	0
*Sarracenia purpurea* subsp. *venosa* var. *burkei* 34	3	1	3	0
*Sarracenia purpurea* subsp. *venosa* var. *burkei* 7	5	0	5	0
*Sarracenia purpurea* subsp. *venosa* var. *burkei* 39	0	0	10	5
*Sarracenia purpurea* subsp. *venosa* var. *burkei* f. *luteola* 48	4	0	2	0
*Sarracenia purpurea* subsp. *venosa* var. *montana* 41	11	1	0	0
*Sarracenia purpurea* subsp. *venosa* var. *montana* 9	13	0	2	1
*Sarracenia rubra* subsp. *alabamensis* 2	2	0	7	1
*Sarracenia rubra* subsp. *gulfensis* 25	6	0	7	0
*Sarracenia rubra* subsp. *jonesii* 3	3	0	7	2
*Sarracenia rubra* subsp. *wherryi* 23	12	3	11	4
Average	6,4	1,0	6,6	1,1

The pitcher dataset contained 589 compounds detected in at least one sample. Among these, there were library matches (≥ 70%) for 67 alcohols, 60 aldehydes and ketones, 72 esters, 60 ethers, 52 carboxylic acids and sterols, 50 hydrocarbons (including those identified as alkanes), 139 n-alkanes, 74 nitrogen-containing compounds and 15 sulphur-containing compounds. Each individual plant's pitcher sample had an average of 48 compounds. The pitcher sample *S*. *purpurea* subsp. *venosa* var. *montana* (41) did not contain a single compound at a detectable concentration level and *S*. *leucophylla* var. *alba* (26) had the highest number of compounds (n = 78). The barplot in S5 Fig shows the distribution of compounds across all the pitcher samples. Furthermore, every pitcher sample had approximately seven unique compounds, one of which, on average, can be considered as a floral scent component [29]. *Sarracenia flava* var. *atropurpurea* (31) and *S*. *minor* var. *okefenokeensis* (5) had the highest number (n = 6) of floral scent compounds (Table 3). Four samples, *S*. *leucophylla* (12), *S*. *psittacina* f. *heterophylla* (6), *Sarracenia purpurea* subsp. *purpurea* (16) and *S*. *purpurea* subsp. *venosa* var. *montana* (41) did not contain unique compounds, whereas *S*. *flava* var. *atropurpurea* (31) had the highest number of unique compounds (n = 25). S1B Table shows the compounds unique to each sample along with their concentration levels. Similar to the lids, pitcher pairs had an average of 32 unique compounds (S2B Table).

A sarracenin-like compound was found at an elution time of 18.2 min. Its mass peak was *m/z* 225, major fragments *m/z* 180 and 138, and further fragments were *m/z* 162, 120, 93, 67 and 43.

### Selection of metabolites

Overall, both the lid and pitcher datasets are very sparse, with 91.35% zeros in the lid dataset and 91.86% in the pitcher dataset. These datasets are also high dimensional, as described above, with 560 and 589 compounds, respectively, in the lid and pitcher datasets. We performed sparse hierarchical clustering of the data in order to reduce the dimensionality of the datasets and identify the compounds important for clustering. The metabolite features selected using the qualitative and quantitative formats of the data are visualized as heat maps (S6–S9 Figs).

### Integration of phylogenetic clustering

The MP-EST accession tree presented in [2] was integrated with metabolite profiling data. Firstly, the selected metabolite features were visualized as heat maps with the MP-EST accession tree (Fig 1A and Fig 2A, S1A and S2A Figs). Since the best bijective map between the samples of the two studies was selected for these visualizations, six samples from our compound dataset are omitted from each of the heat maps (Fig 1A and Fig 2A, S1A and S2A Figs). Secondly, the MP-EST accession tree was used to assess whether the metabolite profiles support the clade-level classification of the plant family. This was done by comparing the aWCDs against aBCDs as well as the background distance distribution formed by the aSLDs. The aWCDs were lower than aBCDs (Fig 1 and Fig 2, S1 and S2 Figs), indicating that the compound data was consistent with the clade-level classification. From the qualitative data of lids, all aWCDs were less than the mean and median values of the aBCDs. In comparison to the background distribution, eight out of nine aWCDs were less than the mean of the aSLDs and all the aWCDs were less than the median of the aSLDs (Fig 2B). Finally, the aWCDs were significantly lower than the aBCDs (Wilcoxon test *P*-value = 1.42e-05; Fig 2C). From the qualitative data of pitchers, all aWCDs were less than the mean and median values of the aBCDs as well as the aSLDs (Fig 2B), and the aWCDs were significantly lower than aBCDs (*P*-value = 5.109e-06; Fig 2C). The quantitative data weakly supported the clade-level classification (S1 and S2 Figs). From the quantitative data of lids, seven out of nine aWCDs were lower than the mean and median values of the aBCDs and aSLDs (S1B Fig), and the difference between aWCDs and aBCDs was marginally significant (*P*-value = 0.02; S1C Fig. From the quantitative data of pitchers, all aWCDs were lower than the mean of aBCDs, eight out of nine aWCDs were lower than the mean of aSLDs, seven aWCDs were less than the median of aBCDs, and six aWCDs were less than the median of aSLDs (S2B Fig). The difference between aWCDs and aBCDs was marginally significant (*P*-value = 0.004; S2C Fig).

## Discussion

### Coniine in *Sarracenia* sp.

The presence of coniine has been reported from poison hemlock and twelve *Aloe* species [22,23]. The only report of coniine in Sarraceniaceae is by Mody et al. [21], who isolated 5 mg of coniine from 45 kg fresh pitchers of *S*. *flava* via steam distillation. This is in contrast to the results of Romeo et al. [11], who did not detect any alkaloids or volatile amines in *Sarracenia*. We have now confirmed the findings of Mody et al. [21] and also found that coniine occurs, often in low amounts, in at least seven other species, *e*.*g*. *S*. *purpurea* (Table 2). It remains unknown where exactly coniine is biosynthesized in *Sarracenia* spp., since the compound was detected both in lids and in the actual pitchers. Biosynthesis of coniine has been studied in poison hemlock. In this case the carbon backbone is derived from the iterative coupling of butyryl-CoA and two malonyl-CoAs by a PKS, CPKS5 [24]. According to our analysis, genes encoding such enzymes are present in the transcriptomes [32] of *S*. *psittacina* and *S*. *purpurea*. Both species harbour three contigs which represent two to three PKSs. The exact number could not be determined because the N-terminal contig cannot be assigned to either of the C-terminal contigs. The contigs do not represent full-length sequences and therefore it is impossible to clearly assign them as PKSs for coniine biosynthesis in *Sarracenia* spp. Important mutations might be located outside the observed area, preventing distinction from chalcone synthases involved in anthocyanin synthesis [9,10].

An important question is the function of coniine in *Sarracenia*. Why should plants living in nutrient-poor environments produce a nitrogenous compound if there are no benefits? Butler and Ellison [39] studied nitrogen acquisition of *S*. *purpurea* and reported that the pitchers are in fact very efficient in prey capture and could thus greatly enhance the available nitrogen for the following growth season. Mody et al. [21] postulated that coniine could be an insect-stunning agent. Coniine did indeed paralyze fire ants, but probably the tested concentrations were not physiological [21]. Another function for coniine could be insect attraction, as suggested by Harborne [25] and Roberts [40], who identified coniine as a floral scent compound in poison hemlock. In conclusion, it appears that an investment in coniine biosynthesis could have a double benefit by enhancing both insect attraction and retention.

### Metabolite profiles of *Sarracenia* and *Darlingtonia*

There are several previous reports on *Sarracenia* volatiles [7,8]. For example, Miles et al. [7] reported benzothiazole, benzyl alcohol, heptadecane and tridecane from *S*. *flava*, which we also found from *Sarracenia* spp. Nonanal, a floral scent compound widespread in the plant kingdom [28], was found from *Sarracenia* spp. lids in our study. The compound is known to attract mosquitos [41], and Miles et al. [7] described it as one of *S*. *flava*’s volatile organic compounds. The Venus flytrap (*Dionaea muscipula*), another carnivorous plant, emits this volatile organic compound when it is feeding on fruit flies (*Drosophila melanogaster*) [35].

**Fig 4:**
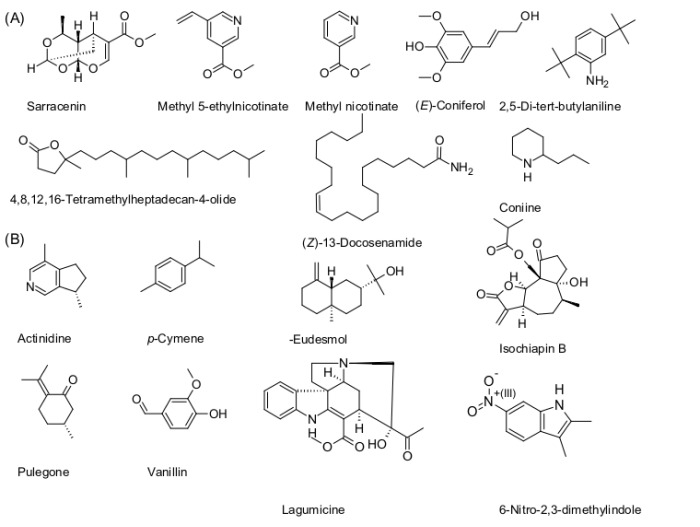
Compounds identified in *Sarracenia* and *D*. *californica*. (A) Common and (B) specific constituents of *Sarracenia* and *D*. *californica*.

Sarracenin (Fig 4A) has previously been reported from *S*. *flava* [17], *S*. *alata*, *S*. *leucophylla*, *S*. *minor* and *S*. *rubra* [18]. Our study confirmed the presence of this compound in all the aforementioned species, except *S*. *minor*, and revealed several new species containing sarracenin, namely, *S*. *psittacina*, *S*. *purpurea* and *D*. *californica*. The compound is volatile and attracts insects to *Heliamphora* sp. [4]. A possible explanation of why *S*. *minor* did not accumulate sarracenin in our study could be that our samples were not feeding on insects at the time of collection, and as a result, they did not synthesize the compound [4].

We also found (*Z*)-13-docosenamide (erucamide) to be a common compound in *Sarracenia* spp. and *D*. *californica*. It has previously been reported from *H*. *tatei* and *H*. *heterodoxa* [4], where it is a possible lubricating component of the nectar.

Other common compounds from *Sarracenia* sp. and *D*. *californica* are carboxylic acids (fatty acids) such as tetradecanoic, hexadecanoic and (*Z*)-9-hexadecenoic acids. All three are floral scent compounds and the latter is known from *Hydnora africana* [42]. Hexadecanoic acid is emitted by the Venus fly trap as a volatile organic compound after feeding [35].

*Sarracenia* spp. display a huge variety of unique compounds which are found only in their lid and/or pitcher. Actinidine is a floral scent compound known from *Sauromatum guttatum* [43] and an insect pheromone in Hymenoptera [44]. *Trans*-Jasmone acts either as an insect attractant or repellent depending on the insect species. Pulegone (Fig 4B) is a floral scent compound of *Tilia* sp. [45] and *Agastache* sp. [46], and functions as an insecticide [47]. 14-β-Pregna is a sex pheromone of the insect *Eurygaster maura* [48]. Lagumicine was found from *S*. *oreophila* lid. Previously it had been found from *Alstonia angustifolia* var. *latifolia* [49]. Miles et al. [17] suggested, on the basis of the possible cleavage of sarracenin, that terpene indole alkaloids could be synthesized in *Sarracenia* spp.

The studied accessions of Sarraceniaceae are characterized by a large number of diverse metabolites, with nearly 600 metabolites identified in lids as well as in pitchers. They are also characterized by a huge chemical diversity, as the metabolite compositions of different plants were largely distinct. Unlike mutation data from highly conserved genomic loci, the data that mainly displays wide heterogeneity of samples is not suitable for constructing taxonomies. Knudsen et al. [29] concluded that the usability of floral scent compounds in chemotaxonomy is limited because chemical composition usually differs even between closely related species. The composition may also vary among genera of a specific family, as it may vary among species of a given genus. Thus, the chemical composition alone is of little use for phylogenetic estimates above the genus level. As expected, clustering derived from our data alone does not agree with the phylogenetic structure of the accessions (see the column dendrograms in S6–S9 Figs).

The available phylogenetic information, on the other hand, may help us to understand the current data. We sought to explain the metabolite composition of plants with the known phylogenetic information from [2]. We successfully demonstrated that the metabolite data conform with the clade-level classification of the plant family and hence that the phylogeny can explain the metabolite composition of the plants to some extent. Notably, whereas the qualitative data could be largely explained by phylogeny (Fig 1 and Fig 2), the concordance of quantitative data with the clade-level classification was relatively weaker (S1 and S2 Figs). Thus, we speculate that evolution may more directly affect the presence or absence of specific chemicals than the exact amount in which the chemicals are present.

We have limited the focus of the current data mining to cataloging and visualizing the data. Given the dominance of zeroes, the current datasets may benefit from computational methods specially designed for zero-inflated or left-censored data. But such a detailed computational analysis is out of the scope of this biochemical profiling study.

## Conclusion

Studied accessions of Sarraceniaceae possessed a diverse variety of compounds. Lids and pitchers were studied separately and approximately 600 compounds were detected in both collections. The accessions also showed huge diversity, with every accession containing unique compounds. Coniine was newly detected in seven *Sarracenia* species in addition to the known source, *S*. *flava*. However, we could not identify a specific candidate gene involved in coniine biosynthesis in *Sarracenia* spp. Among the common constituents of Sarraceniaceae are sarracenin, erucamide, and nonanal. By integrating existing phylogenetic information of Sarraceniaceae, we successfully demonstrated that the phylogeny can explain the metabolite composition of the plants. Phylogeny explained the presence or absence of compounds more strongly than their concentrations.

## Supporting information

S1 Fig Visualization of selected metabolite features from the quantitative data of lids. (A) Heat map visualization of selected metabolite features from the quantitative data of lids. The phylogenetic tree from [2] is displayed as the column dendrogram. Six samples of our dataset (14, 31, 35, 37, 44, and 46) are omitted from this heat map, based on the sample selection procedure described in the Methods section. (B) Comparison of average within-clade distances (aWCDs) against the background distribution of average species-level distances (aSLDs) and average between-clade distances (aBCDs). Distribution of aSLDs was calculated using qualitative data of the selected metabolite features and displayed in a density plot. The black vertical lines mark the individual aWCDs. The orange dashed and dotted lines show the mean and median of aSLDs. The purple dashed and dotted lines show the mean and median of aBCDs. (C) Comparison of aWCDs (green continuous density line) with aBCDs (orange dashed density line).
(PDF)

S2 Fig Visualization of selected metabolite features from the quantitative data of pitchers. (A) Heat map visualization of selected metabolite features from the quantitative data of pitchers. The phylogenetic tree from [2] is displayed as the column dendrogram. Six samples of our dataset (1, 11, 31, 38, 42, and 46) are omitted from this heat map, based on the sample selection procedure described in the Methods section. (B) Comparison of average within-clade distances (aWCDs) against the background distribution of average species-level distances (aSLDs) and average between-clade distances (aBCDs). Distribution of aSLDs was calculated using qualitative data of the selected metabolite features and displayed in a density plot. The black vertical lines mark the individual aWCDs. The orange dashed and dotted lines show the mean and median of aSLDs. The purple dashed and dotted lines show the mean and median of aBCDs. (C) Comparison of aWCDs (green continuous density line) with aBCDs (orange dashed density line).
(PDF)

S3 Fig Alignment of *Sarracenia* PKSs with selected plant-PKSs translated into an amino acid sequence. Conserved amino acids of the active site are bolded, and colored amino acids indicate mutated amino acids of the active site. GenBank accession numbers: *Conium maculatum* CPKS1 (KP726914), *Conium maculatum* CPKS2 (KP726915), *Conium maculatum* CPKS5 (KP726916), *Gerbera hybrida* 2PS (CAA86219.2), *Gerbera hybrida* CHS1 (Z38096.1), *Medicago sativa* CHS2 (L02902.1).
(PDF)

S4 Fig Barplot of distribution of compounds across lid samples. (PDF)

S5 Fig Barplot of distribution of compounds across pitcher samples. (PDF)

S6 Fig Heat map of selected features obtained from qualitative data of lids. (PDF)

S7 Fig Heat map of selected features obtained from quantitative data of lids. (PDF)

S8 Fig Heat map of selected features obtained from qualitative data of pitchers. (PDF)

S9 Fig Heat map of selected features obtained from quantitative data of pitchers. (PDF)

S1 Table Unique compounds with their concentration percentages in metabolite samples. Unique compounds found in *Sarracenia* and *D*. *californica* (lids and pitchers separately).
(XLSX)

S2 Table Sample comparison in pairs. Samples are compared to each other in lids and pitchers separately.
(XLSX)
