# Supplementary figures and images for: Metabolite profiling of the carnivorous pitcher plants Darlingtonia and Sarracenia
Source: PLoS One. 2017 Feb 21;12(2):e0171078. doi: 10.1371/journal.pone.0171078 (PMC5319649; doi:10.1371/journal.pone.0171078)

(A)

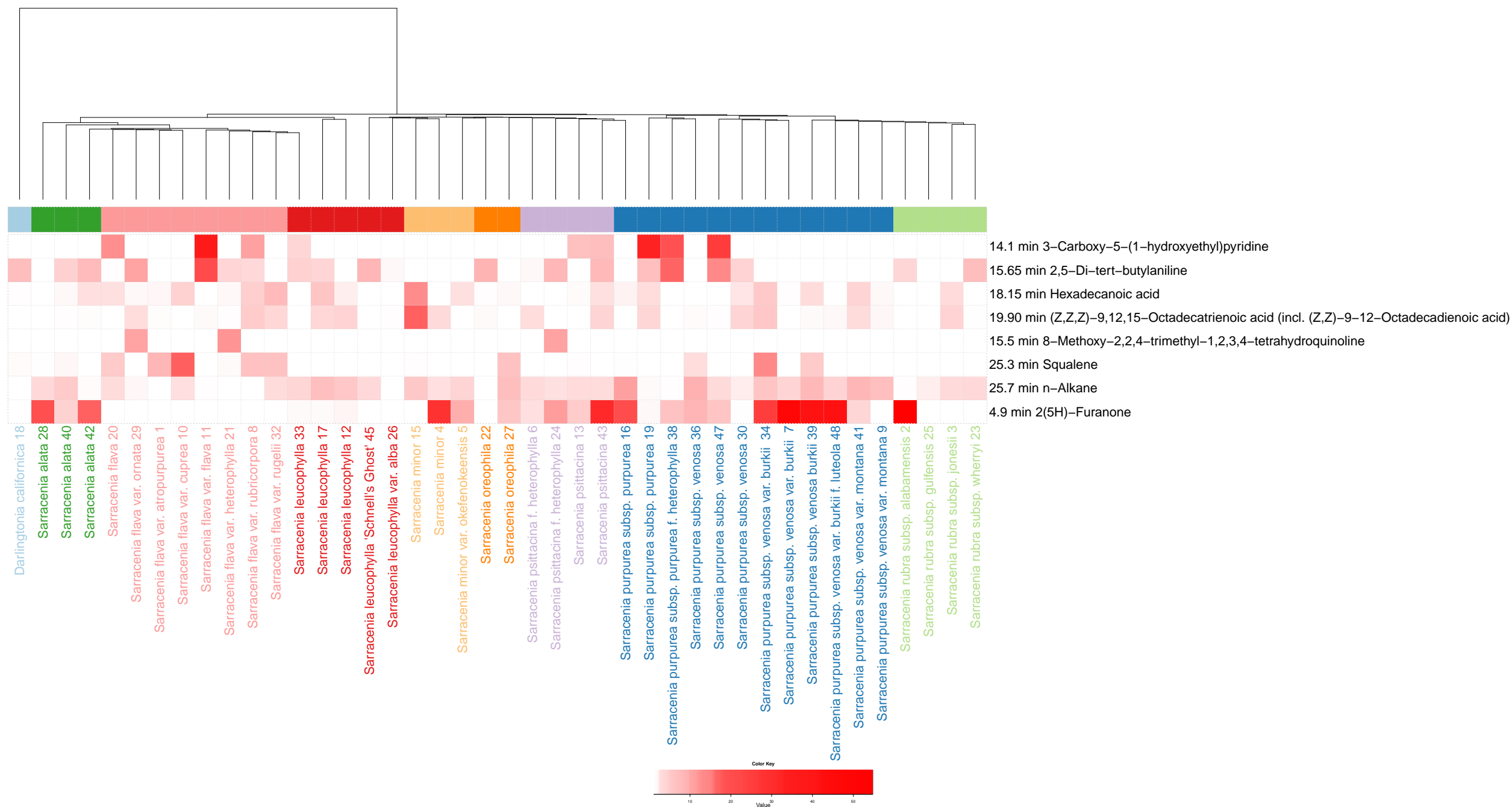

(B)

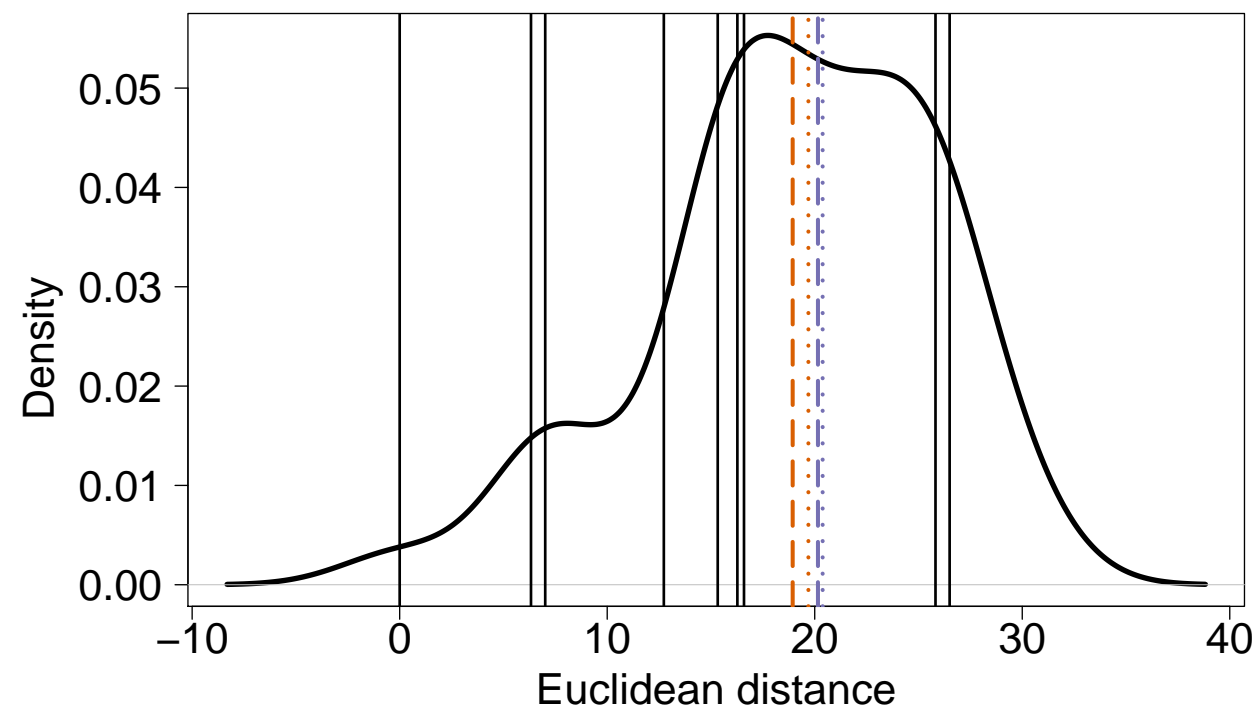

(C)

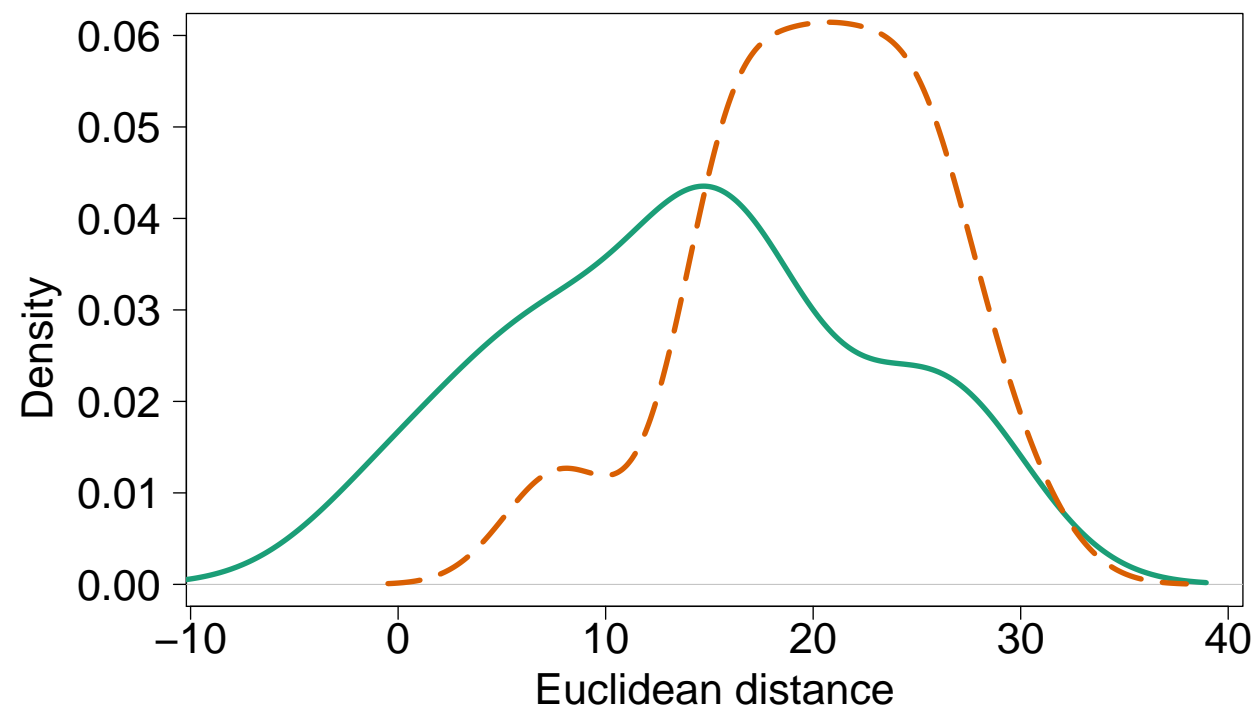

Supplement: S1 Fig — (A) Heat map visualization of selected metabolite features from the quantitative data of lids. The phylogenetic tree from [2] is displayed as the column dendrogram. Six samples of our dataset (14, 31, 35, 37, 44, and 46) are omitted from this heat map, based on the sample selection procedure described in the Methods section. (B) Comparison of average within-clade distances (aWCDs) against the background distribution of average species-level distances (aSLDs) and average between-clade distances (aBCDs). Distribution of aSLDs was calculated using qualitative data of the selected metabolite features and displayed in a density plot. The black vertical lines mark the individual aWCDs. The orange dashed and dotted lines show the mean and median of aSLDs. The purple dashed and dotted lines show the mean and median of aBCDs. (C) Comparison of aWCDs (green continuous density line) with aBCDs (orange dashed density line). (PDF) [file pone.0171078.s001.pdf]

(A)

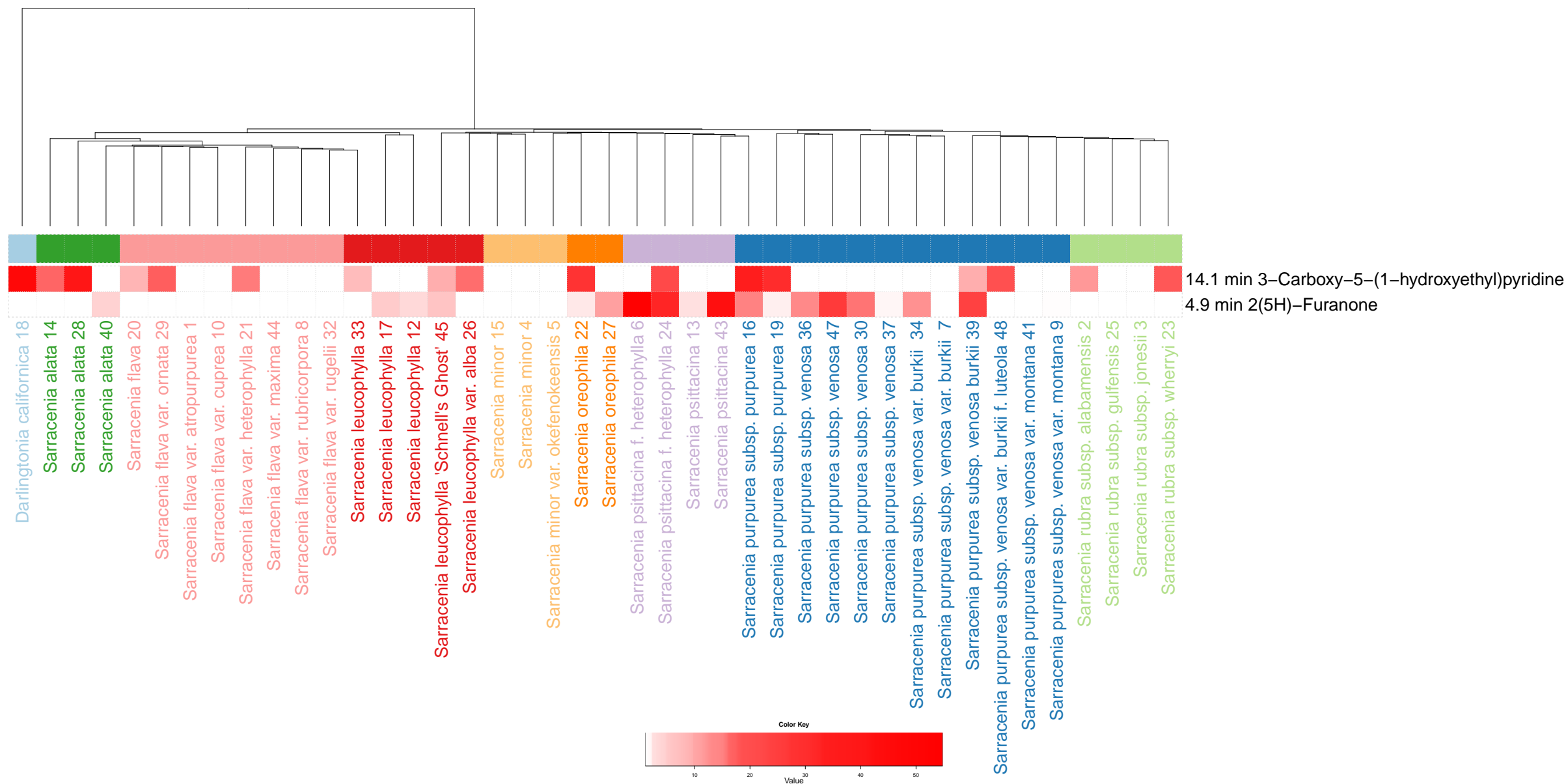

(B)

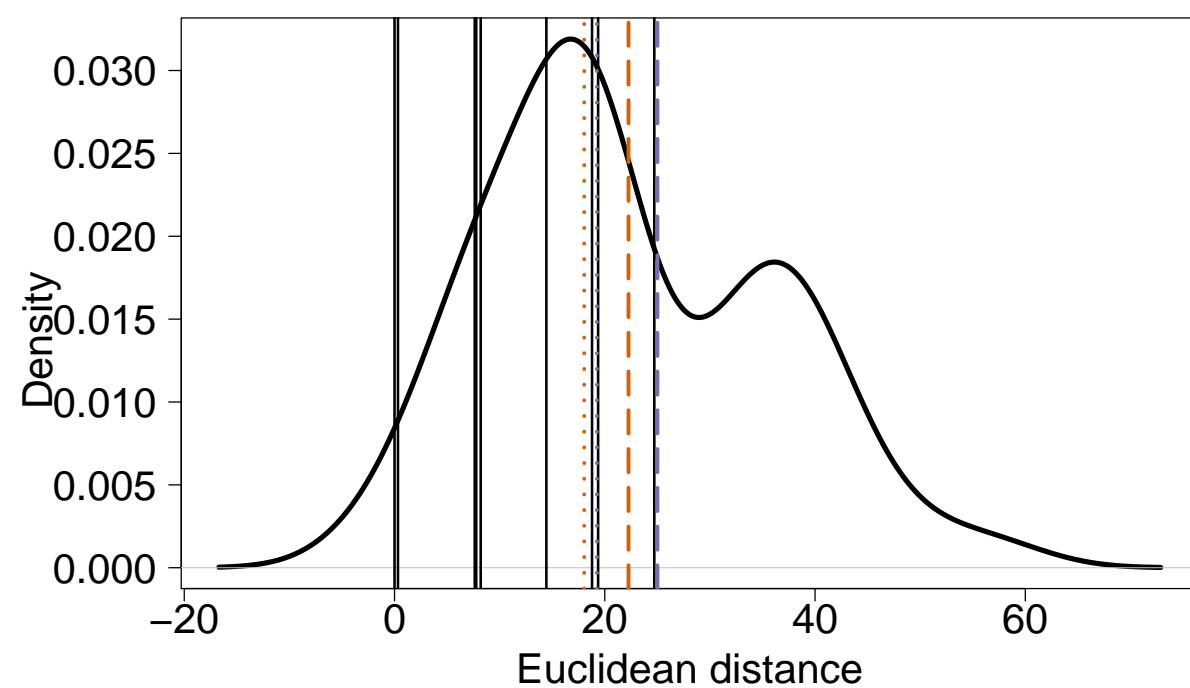

(C)

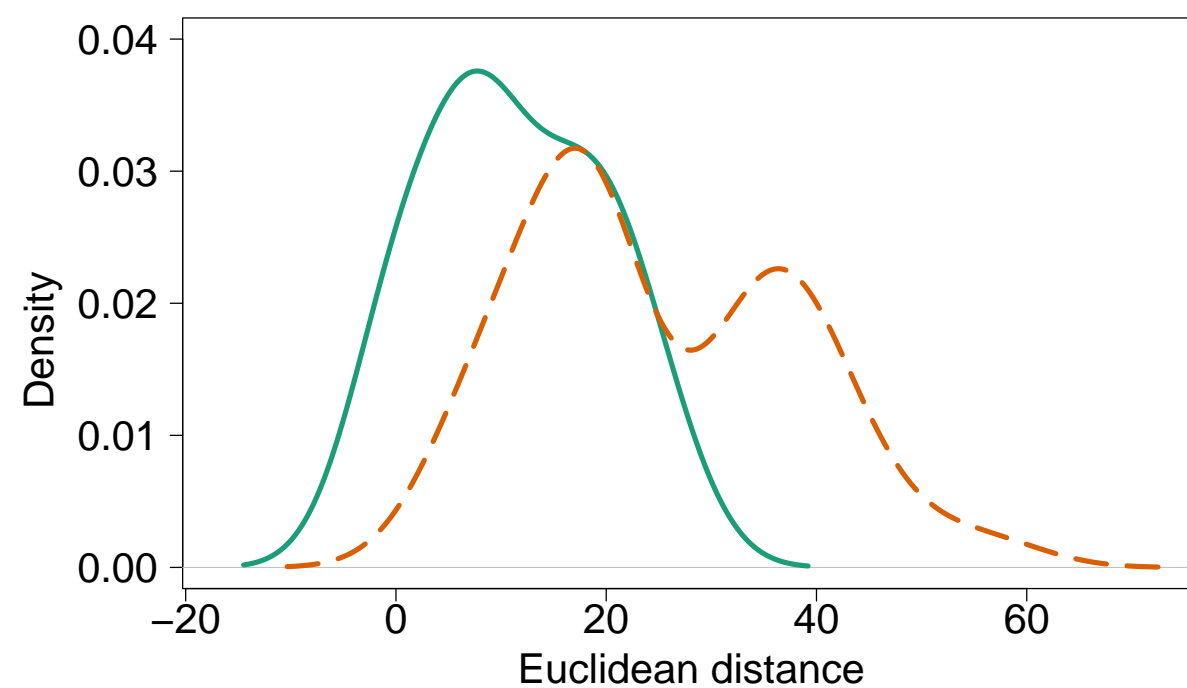

Supplement: S2 Fig — (A) Heat map visualization of selected metabolite features from the quantitative data of pitchers. The phylogenetic tree from [2] is displayed as the column dendrogram. Six samples of our dataset (1, 11, 31, 38, 42, and 46) are omitted from this heat map, based on the sample selection procedure described in the Methods section. (B) Comparison of average within-clade distances (aWCDs) against the background distribution of average species-level distances (aSLDs) and average between-clade distances (aBCDs). Distribution of aSLDs was calculated using qualitative data of the selected metabolite features and displayed in a density plot. The black vertical lines mark the individual aWCDs. The orange dashed and dotted lines show the mean and median of aSLDs. The purple dashed and dotted lines show the mean and median of aBCDs. (C) Comparison of aWCDs (green continuous density line) with aBCDs (orange dashed density line). (PDF) [file pone.0171078.s002.pdf]

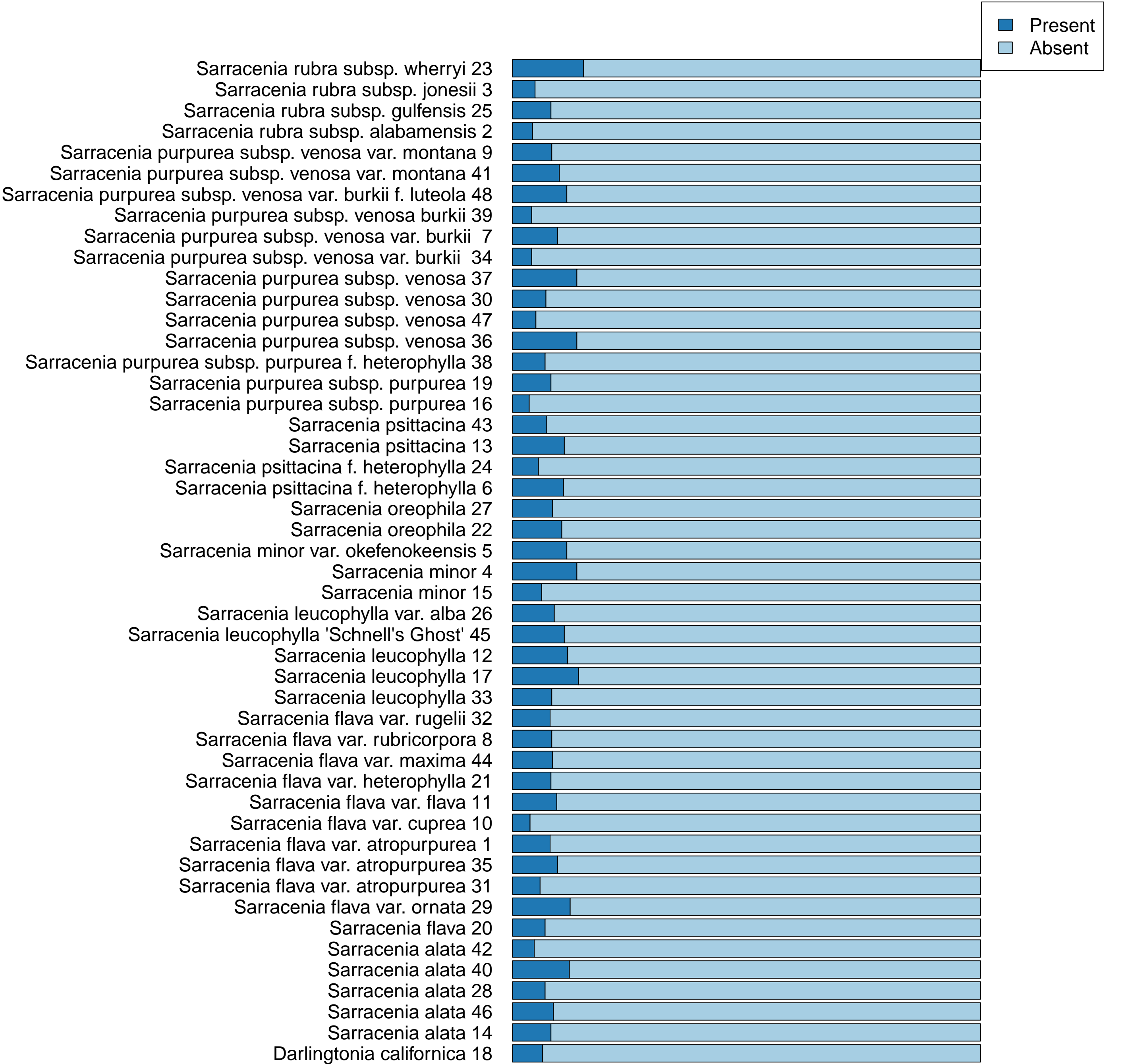

0 100 200 300 400 500

Supplement: S4 Fig — (PDF) [file pone.0171078.s004.pdf]

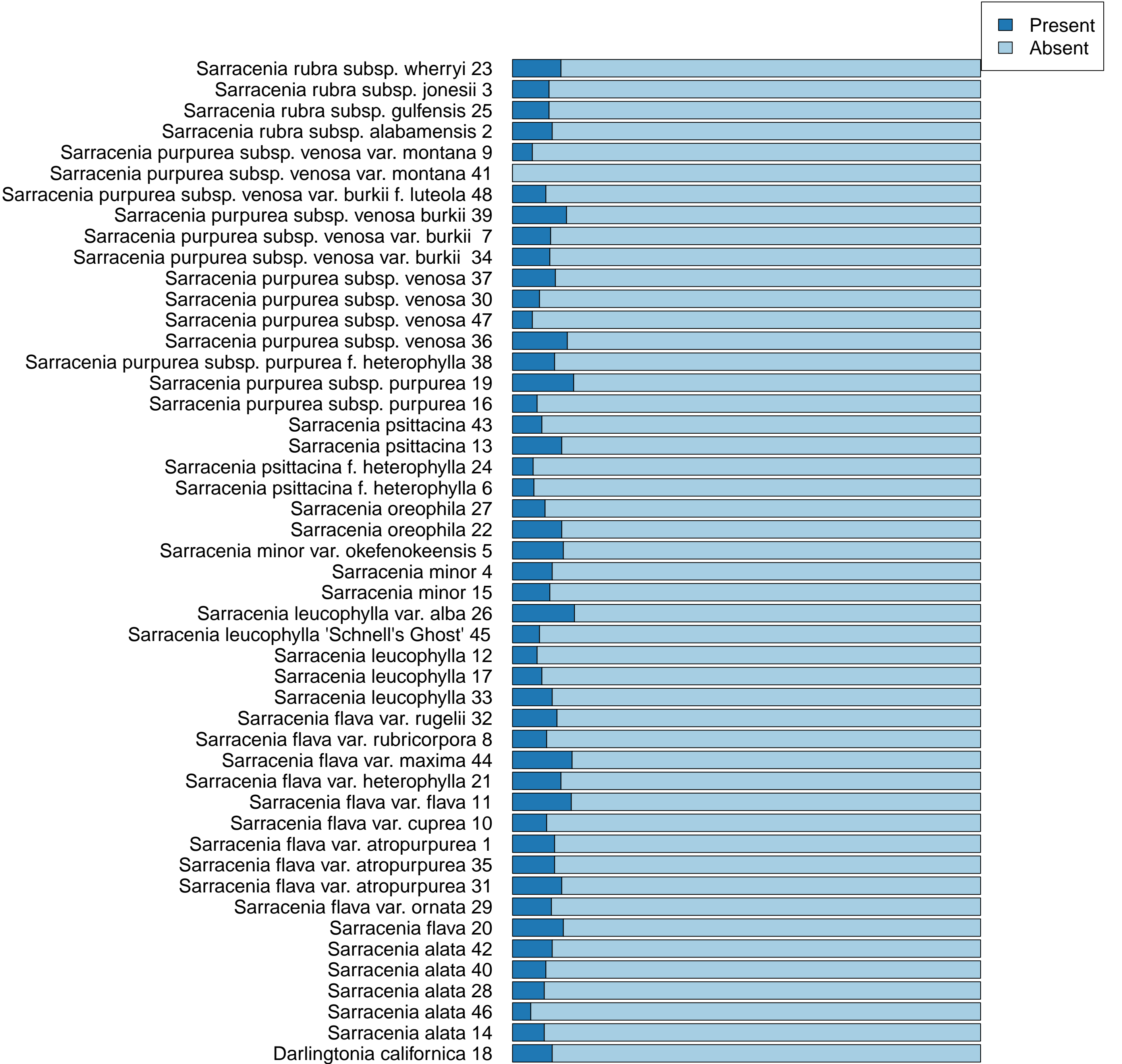

0 100 200 300 400 500

Supplement: S5 Fig — (PDF) [file pone.0171078.s005.pdf]

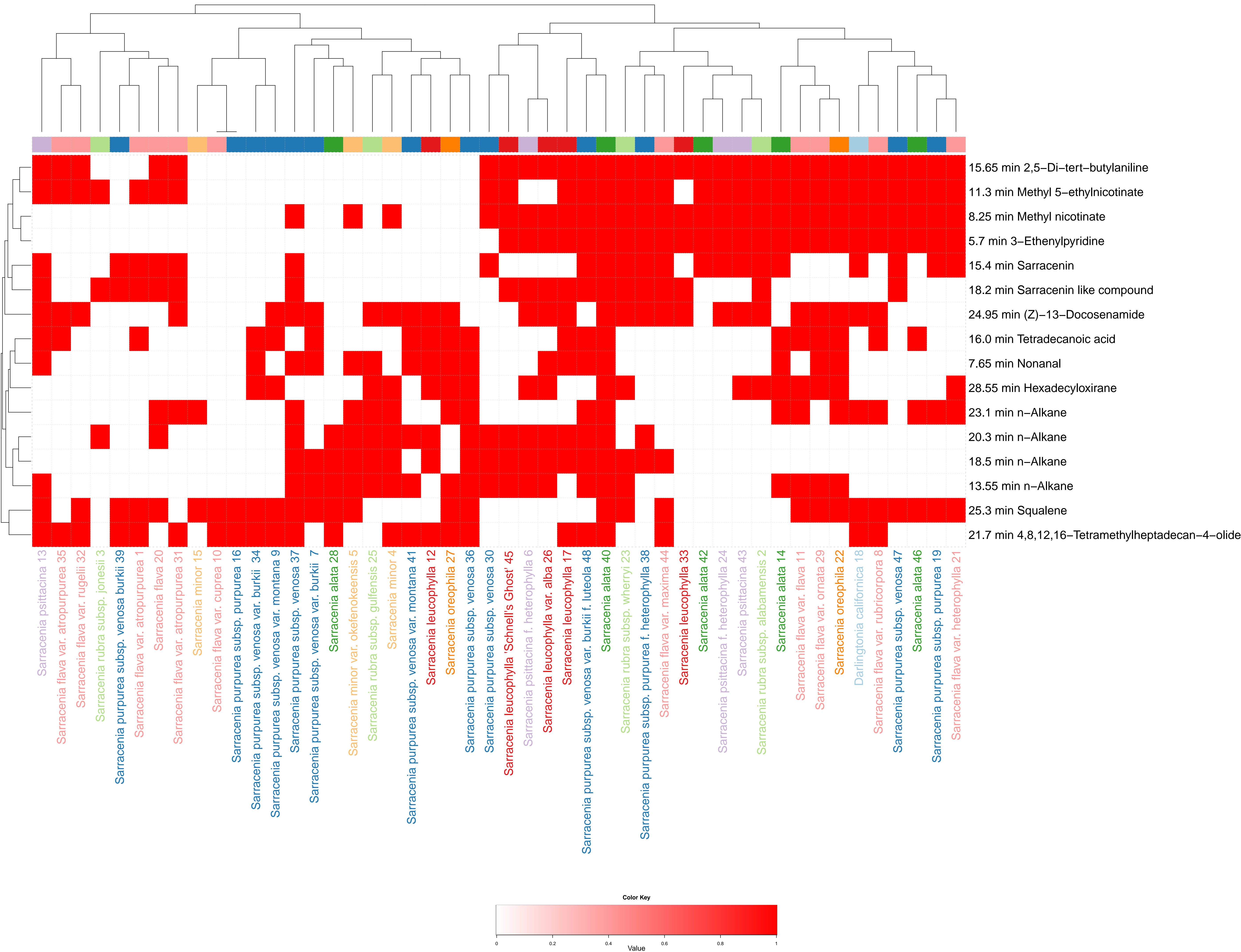

Supplement: S6 Fig — (PDF) [file pone.0171078.s006.pdf]

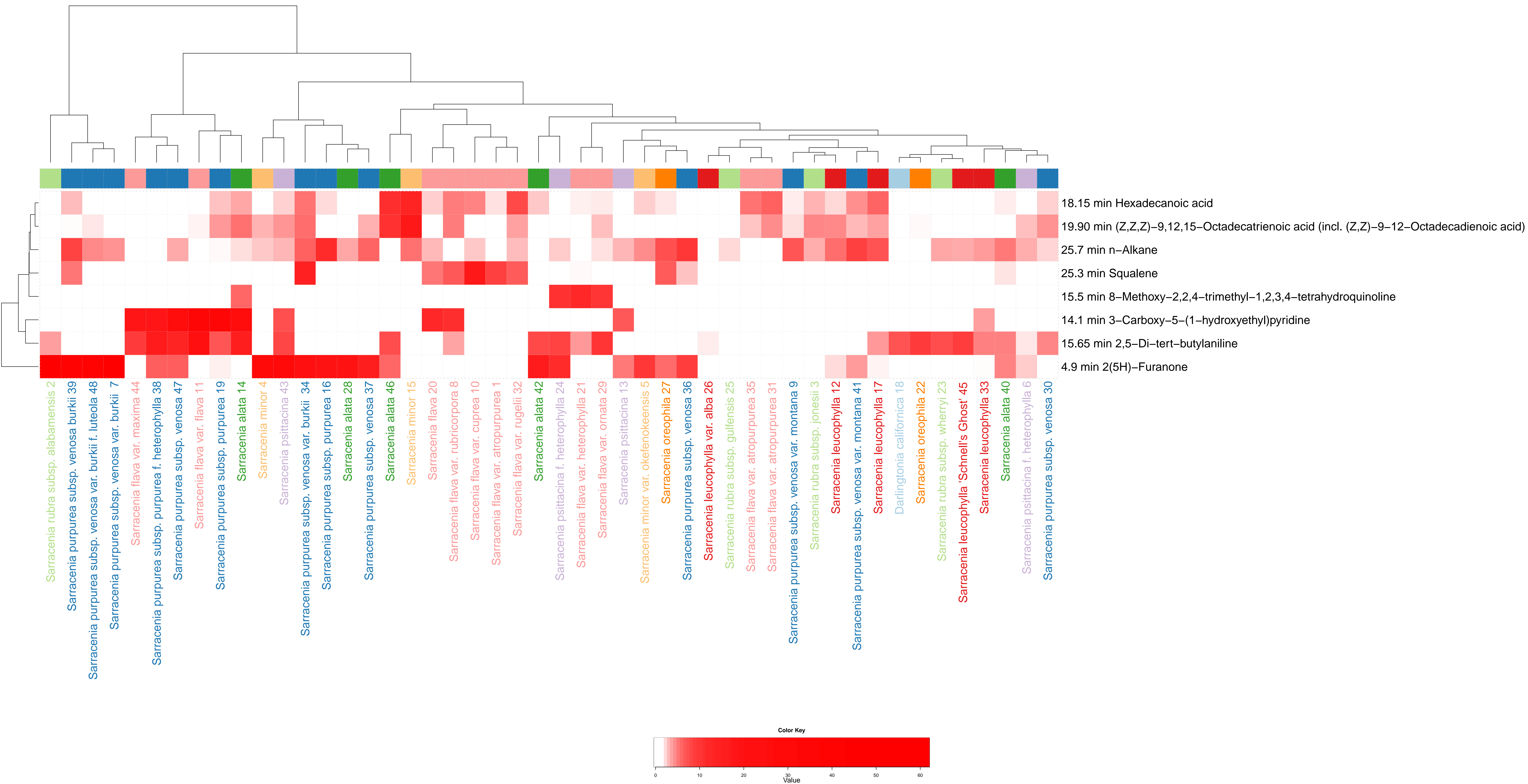

Supplement: S7 Fig — (PDF) [file pone.0171078.s007.pdf]

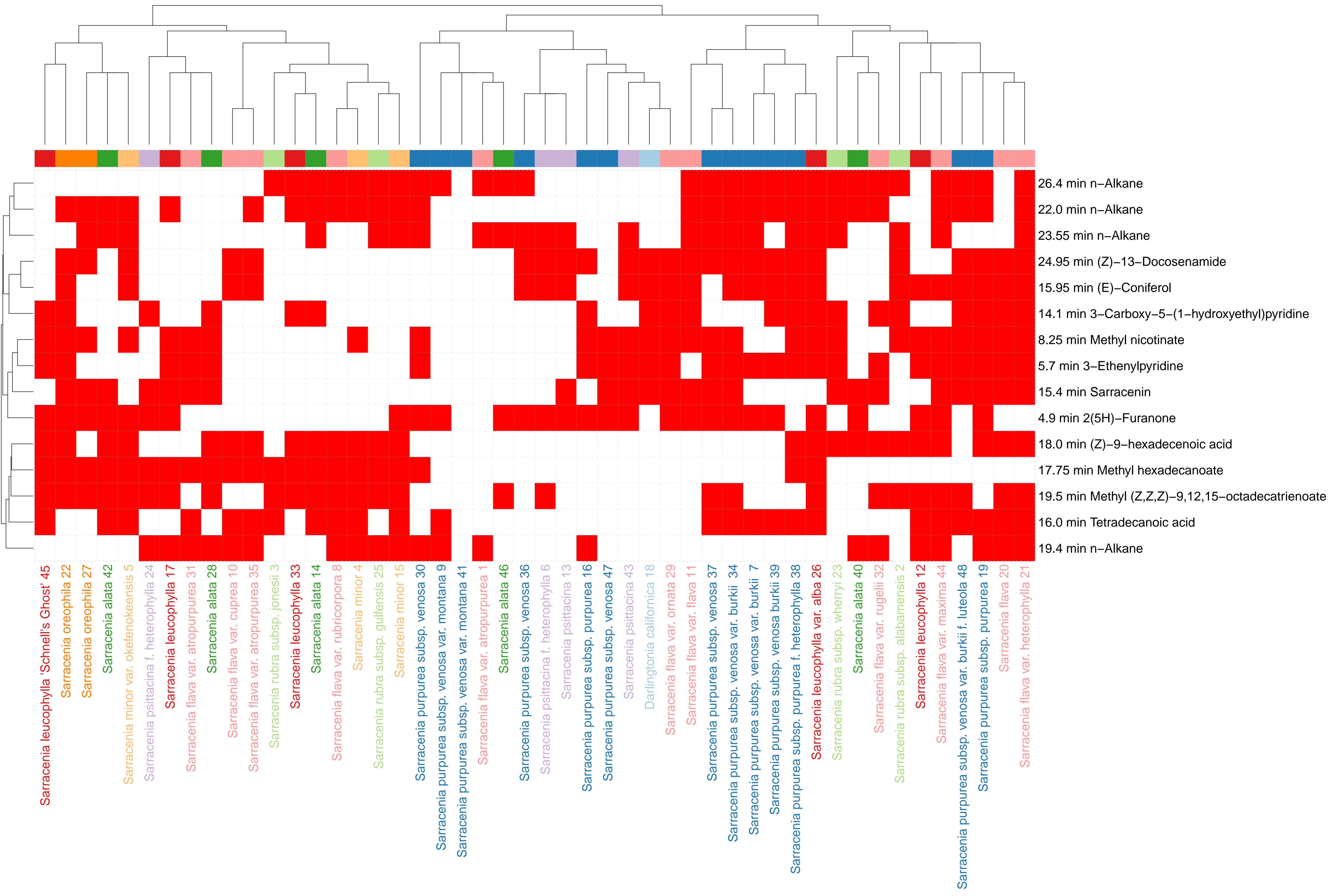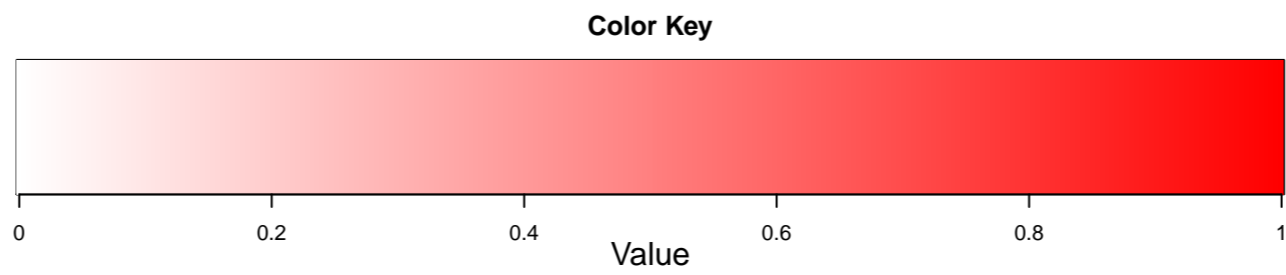

Supplement: S8 Fig — (PDF) [file pone.0171078.s008.pdf]

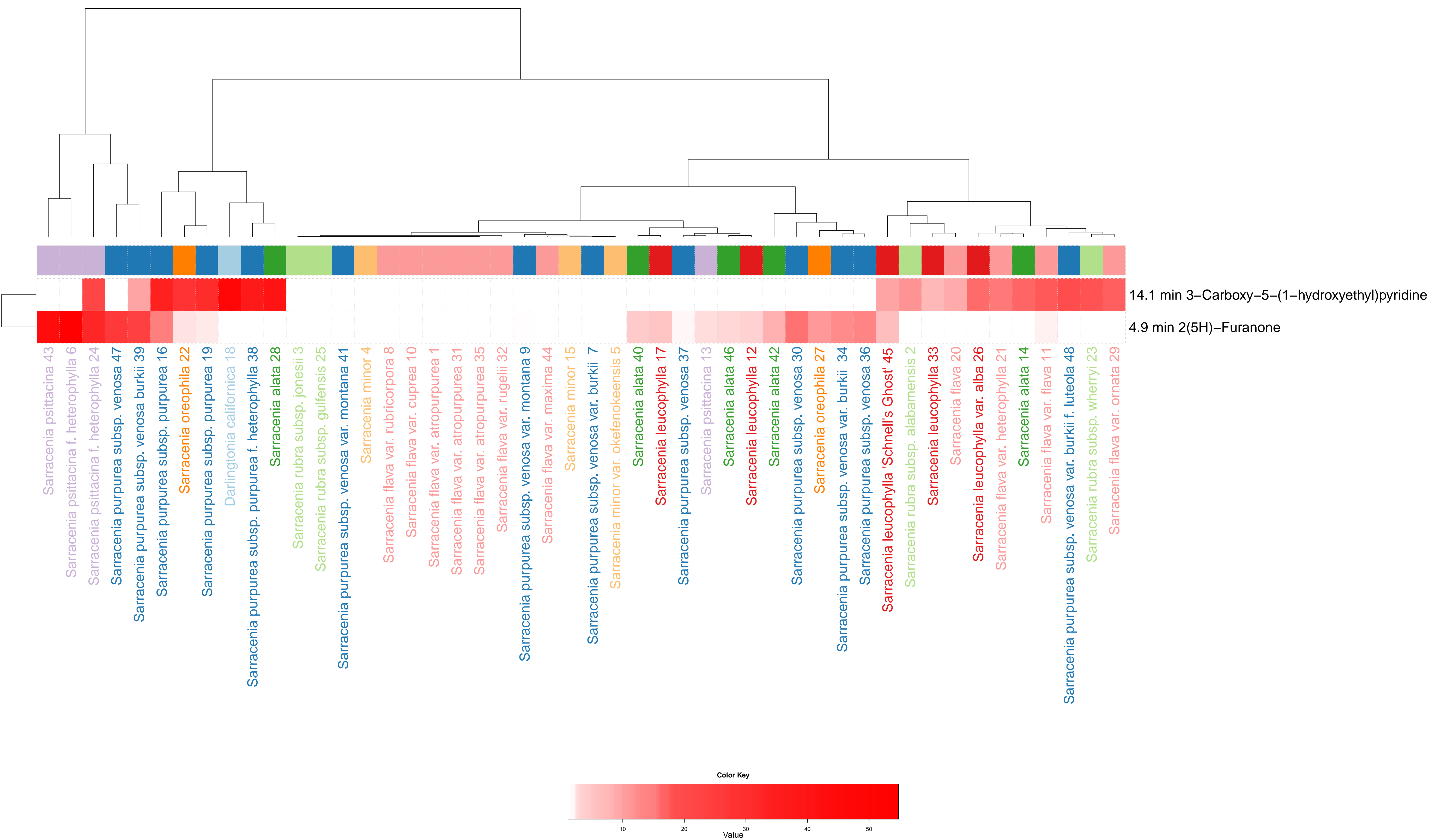

Supplement: S9 Fig — (PDF) [file pone.0171078.s009.pdf]
