# Supplementary material for: Metabolite profiling of the carnivorous pitcher plants Darlingtonia and Sarracenia
Source: PLoS One. 2017 Feb 21;12(2):e0171078. doi: 10.1371/journal.pone.0171078 (PMC5319649; doi:10.1371/journal.pone.0171078)
Supplement: S3 Fig — Conserved amino acids of the active site are bolded, and colored amino acids indicate mutated amino acids of the active site. GenBank accession numbers: Conium maculatum CPKS1 (KP726914), Conium maculatum CPKS2 (KP726915), Conium maculatum CPKS5 (KP726916), Gerbera hybrida 2PS (CAA86219.2), Gerbera hybrida CHS1 (Z38096.1), Medicago sativa CHS2 (L02902.1). (PDF) [file pone.0171078.s003.pdf]

```
                                1        10        20        30        40        50        60
                                |        |         |         |         |         |         |
Conium PKS5                     -----MVTVNEFRKAHLAEGPATVLAIGTATPSYCIDQSTFPDLYFRTTKSEDETELKEK
Medicago CHS2                   -----MVSVSEIRKAQRAEGPATILAIGTANPANCVEQSTYPDFYFKITNSEHKTELKEK
Conium PKS2                     -----MVTVNEFRKAQQAEGPATVLAIGTATPPNCVDQSTYADYYFRVTKSEDKTELKEK
Gerbera CHS1                    --MASSVDMKAIRDAQRAEGPATILAIGTATPANCVYQADYPDYYFRITKSEHMVDLKEK
Conium PKS1                     -MANHSAKIEEIRKTQRAQGPANVLAIGTATPSNCVYQADYPDYHFRITNSKHMTDLKLK
Gerbera 2PS                     MGSYSSDDVEVIREAGRAQGLATILAIGTATPPNCVAQADYADYYFRVTKSEHMVDLKEK
Spsittacina-10591_isotig10557   -----MVSVDEVRKAQRAEGPATVMAIGTATPPNCVDQSTYPDYYFRITNSEHKAELKEK
Spsittacina-26038_isotig10557   -----MVSVDEVRKAQRAEGPATVMAIGTATPPNCVDQSTYPDYYFRITNSEHKAELKEK
Spurpurea-22109_isotig09797     -----MVTVEEVRKAQRAEGPATVLAIGTATPPNCVDQSTYPDYYFRITNSEHKTELKEK
Spurpurea-9803_isotig09797      -----MVTVEEVRKAQRAEGPATVLAIGTATPPNCVDQSTYPDYYFRITNSEHKTELKEK
Spurpurea-475_isotig00474       ------------------------------------------------------------
Spurpurea-12781_isotig00474     ------------------------------------------------------------
Spurpurea-478_isotig00477       ------------------------------------------------------------
Spurpurea-12783_00476           ------------------------------------------------------------
Spurpurea-477_isotig00476       ------------------------------------------------------------
Spurpurea-476_isotig00475       ------------------------------------------------------------
Spurpurea-12782_isotig00475     ------------------------------------------------------------
Spurpurea-12784_isotig00477     ------------------------------------------------------------
Spsittacina-5295_isotig05263    ------------------------------------------------------------
Spsittacina-5294_isotig05262    ------------------------------------------------------------
Spsittacina-20742_isotig05263   ------------------------------------------------------------
Spsittacina-20741_isotig05262   ------------------------------------------------------------
Spurpurea-4182_isotig04177      ------------------------------------------------------------
Spurpurea-16488_isotig04177     ------------------------------------------------------------
Spsittacina-24513_isotig09032   ------------------------------------------------------------
Spsittacina-9066_isotig09032    ------------------------------------------------------------

                                61       70        80        90        100       110       120
                                |        |         |         |         |         |         |
Conium PKS5                     FKRMCDRSMINTRYTHLTEEFMKENPDFWN-MAPSLDARQEIVVNEVPKLGKEAATKAIK
Medicago CHS2                   FQRMCDKSMIKRRYMYLTEEILKENPNVCEYMAPSLDARQDMVVVEVPRLGKEAAVKAIK
Conium PKS2                     FKRMCDKSMISTRYMHLTEELLKENPEICEYMAPSLDARQDIVVNEVPKLGKEAATRAIK
Gerbera CHS1                    FKRMCDKSMIRKRYMHITEEYLKQNPNMCAYMAPSLDVRQDLVVVEVPKLGKEAAMKAIK
Conium PKS1                     FKRMCEKSMIRKRYMHLTKDYLKKNPNVCAYEAPSLDARQDLVVVEVPRLGKEAASKAIK
Gerbera 2PS                     FKRICEKTAIKKRYLALTEDYLQENPTMCEFMAPSLNARQDLVVTGVPMLGKEAAVKAID
Spsittacina-10591_isotig10557   FQRMCDKSMIKKRYMYLTEEILKQNPNVCAYMAPSLDARQDMVVVEVPKLGKDAAVKAIK
Spsittacina-26038_isotig10557   FQRMCDKSMIKKRYMYLTEEILKQNPNVCAYMAPSLDARQDMVVVEVPKLGKDAAVKAIK
Spurpurea-22109_isotig09797     FKRMCKKKHI---YIFLYMYILIDIYLICMF-----------------------------
Spurpurea-9803_isotig09797      FKRMCKKKHI---YIFLYMYILIDIYLICMF-----------------------------
Spurpurea-475_isotig00474       ------------------------------------------------------------
Spurpurea-12781_isotig00474     ------------------------------------------------------------
Spurpurea-478_isotig00477       ------------------------------------------------------------
Spurpurea-12783_isotig00476     ------------------------------------------------------------
Spurpurea-477_isotig00476       ------------------------------------------------------------
Spurpurea-476_isotig00475       ------------------------------------------------------------
Spurpurea-12782_isotig00475     ------------------------------------------------------------
Spurpurea-12784_isotig00477     ------------------------------------------------------------
Spsittacina-5295_isotig05263    ------------------------------------------------------------
Spsittacina-5294_isotig05262    ------------------------------------------------------------
Spsittacina-20742_isotig05263   ------------------------------------------------------------
Spsittacina-20741_isotig05262   ------------------------------------------------------------
Spurpurea-4182_isotig04177      ------------------------------------------------------------
Spurpurea-16488_isotig04177     ------------------------------------------------------------
Spsittacina-24513_isotig09032   ------------------------------------------------------------
Spsittacina-9066_isotig09032    ------------------------------------------------------------

                                121      130       140       150       160       170       180
                                |        |         |         |         |         |         |
Conium PKS5                     EWGQPMSKITHLVFCTYSSADFPGADFRLTKLLGLSPSVKRSMLYQQGCFAGGTGLRLAK
Medicago CHS2                   EWGQPKSKITHLIVCTTSGVDMPGADYQLTKLLGLRPYVKRYMMYQQGCFAGGTVLRLAK
Conium PKS2                     EWGQPKSKITHLIFCTTSGVDMPGADFRLTKLLGLRPSVKRFMLYQQGCFAGGTVLRLAK
Gerbera CHS1                    EWGHPKSKITHLIFCTTSGVDMPGADYQLTKLLGLRPSVKRFMMYQQGCFAGGTVLRLAK
Conium PKS1                     EWGQSKSKITHLIFCTTSGVDMPGADYQLTKLLGLRPSVKRFMMYQQGCFAGGTVLRLAK
Gerbera 2PS                     EWGLPKSKITHLIFCTTAGVDMPGADYQLVKLLGLSPSVKRYMLYQQGCAAGGTVLRLAK
Spsittacina-10591_isotig10557   EWGQPKSKITHLVF----------------------------------------------
Spsittacina-26038_isotig10557   EWGQPKSKITHLVF----------------------------------------------
Spurpurea-22109_isotig09797     ------------------------------------------------------------
Spurpurea-9803_isotig09797      ------------------------------------------------------------
Spurpurea-475_isotig00474       ------------------------------------------------------------
Spurpurea-12781_isotig00474     ------------------------------------------------------------
Spurpurea-478_isotig00477       ------------------------------------------------------------
Spurpurea-12783_isotig00476     ------------------------------------------------------------
Spurpurea-477_isotig00476       ------------------------------------------------------------
Spurpurea-476_isotig00475       ------------------------------------------------------------
Spurpurea-12782_isotig00475     ------------------------------------------------------------
```

```
Spurpurea-12784_isotig00477          ------------------------------------------------------------
Spsittacina-5295_isotig05263         ------------------------------------------------------------
Spsittacina-5294_isotig05262         ------------------------------------------------------------
Spsittacina-20742_isotig05263        ------------------------------------------------------------
Spsittacina-20741_isotig05262        ------------------------------------------------------------
Spurpurea-4182_isotig04177           ------------------------------------------------------------
Spurpurea-16488_isotig04177          ------------------------------------------------------------
Spsittacina-24513_isotig09032        ------------------------------------------------------------
Spsittacina-9066_isotig09032         ------------------------------------------------------------

                                     181      190       200       210       220       230       240
                                     |        |         |         |         |         |         |
Conium PKS5                          DLAENNKGARVLVVCSELSVLAFQGPKVIDIDCLITQALFGDGAVAVIVGSDPVIGVEKP
Medicago CHS2                        DLAENNKGARVLVVCSEVTAVTFRGPSDTHLDSLVGQALFGDGAAALIVGSDPVPEIEKP
Conium PKS2                          DLAENNKGARVLIVCSEITVVTFRGPNDTHLDSLVGQALFGDGAAAVIVGSDPAIGIEKP
Gerbera CHS1                         DLAENNKGARVLVVCSEITAVTFRGPNDTHLDSLVGQALFGDGAAAVIVGSDPDLTTERP
Conium PKS1                          DLAENNAGARVLVVCSEITAVTFRGPSDSHLDSLVGQALFGDGAAAVIVGSDPDLSVERP
Gerbera 2PS                          DLAENNKGSRVLIVCSEITAILFHGPNENHLDSLVAQALFGDGAAALIVGSGPHLAVERP
Spsittacina-10591_isotig10557        ------------------------------------------------------------
Spsittacina-26038_isotig10557        ------------------------------------------------------------
Spurpurea-22109_isotig09797          ------------------------------------------------------------
Spurpurea-9803_isotig09797           ------------------------------------------------------------
Spurpurea-475_isotig00474            ------------------------------------------------------------
Spurpurea-12781_isotig00474          ------------------------------------------------------------
Spurpurea-478_isotig00477            ------------------------------------------------------------
Spurpurea-12783_isotig00476          ------------------------------------------------------------
Spurpurea-477_isotig00476            ------------------------------------------------------------
Spurpurea-476_isotig00475            ------------------------------------------------------------
Spurpurea-12782_isotig00475          ------------------------------------------------------------
Spurpurea-12784_isotig00477          ------------------------------------------------------------
Spsittacina-5295_isotig05263         ------------------------------------------------------------
Spsittacina-5294_isotig05262         ------------------------------------------------------------
Spsittacina-20742_isotig05263        ------------------------------------------------------------
Spsittacina-20741_isotig05262        ------------------------------------------------------------
Spurpurea-4182_isotig04177           ------------------------------------------------------------
Spurpurea-16488_isotig04177          ------------------------------------------------------------
Spsittacina-24513_isotig09032        ------------------------------------------------------------
Spsittacina-9066_isotig09032         ------------------------------------------------------------

                                     241      250       260       270       280       290       300
                                     |        |         |         |         |         |         |
Conium PKS5                          LFEIFSAAQTIIPDSDGAIKGYLRKVGLTFHLRKDVPGLIAKNIRKYLVEAFQPLGITDW
Medicago CHS2                        IFEMVWTAQTIAPDSEGAIDGHLREAGLTFHLLKDVPGIVSKNITKALVEAFEPLGISDY
Conium PKS2                          LFEIVSAAQTILPDSDGAIDGHLREVGLTFHLLKDVPGLISKNIRKSLVEAFKPLGITDW
Gerbera CHS1                         LFEMVSAAQTILPDSEGAIDGHLREVGLTFHLLKDVPGLISKNIEKALTTAFSPLGINDW
Conium PKS1                          LFQLISAAQTILPDSDGAIDGHLREVGLTFHLLKDVPGLISKNIEKSLKEAFGPIGISDW
Gerbera 2PS                          IFEIVSTDQTILPDTEKAMKLHLREGGLTFQLHRDVPLMVAKNIENAAEKALSPLGITDW
Spsittacina-10591_isotig10557        ------------------------------------------------------------
Spsittacina-26038_isotig10557        ------------------------------------------------------------
Spurpurea-22109_isotig09797          ------------------------------------------------------------
Spurpurea-9803_isotig09797           ------------------------------------------------------------
Spurpurea-475_isotig00474            ----------------------------TFHLLKDVPGLISKNIEKSLVEAFQPLGISDW
Spurpurea-12781_isotig00474          ----------------------------TFHLLKDVPGLISKNIEKSLVEAFQPLGISDW
Spurpurea-478_isotig00477            ----------------------------TFHLLKDVPGLISKNIEKSLVEAFQPLGISDW
Spurpurea-12783_isotig00476          ----------------------------TFHLLKDVPGLISKNIEKSLVEAFQPLGISDW
Spurpurea-477_isotig00476            ----------------------------TFHLLKDVPGLISKNIEKSLVEAFQPLGISDW
Spurpurea-476_isotig00475            ----------------------------TFHLLKDVPGLISKNIEKSLVEAFQPLGISDW
Spurpurea-12782_isotig00475          ----------------------------TFHLLKDVPGLISKNIEKSLVEAFQPLGISDW
Spurpurea-12784_isotig00477          ----------------------------TFHLLKDVPGLISKNIEKSLVEAFQPLGISDW
Spsittacina-5295_isotig05263         -----------RAHSEGAIDGHLREVGLTFHLLKDVPGLISKNIEKSLVEAFQPLGISDW
Spsittacina-5294_isotig05262         -----------RAHSEGAIDGHLREVGLTFHLLKDVPGLISKNIEKSLVEAFQPLGISDW
Spsittacina-20742_isotig05263        -----------RAHSEGAIDGHLREVGLTFHLLKDVPGLISKNIEKSLVEAFQPLGISDW
Spsittacina-20741_isotig05262        -----------RAHSEGAIDGHLREVGLTFHLLKDVPGLISKNIEKSLVEAFQPLGISDW
Spurpurea-4182_isotig04177           ---------------------------------------------------------SDW
Spurpurea-16488_isotig04177          ---------------------------------------------------------SDW
Spsittacina-24513_isotig09032        --------------------------------------------------RSSHVGISDW
Spsittacina-9066_isotig09032         --------------------------------------------------RSSHVGISDW

                                     301      310       320       330       340       350       360
                                     |        |         |         |         |         |         |
Conium PKS5                          NSIFWIAHPGGPAILDQIEKELSLKPEKLKSSRQILRDYGNLSSASVLFIMDEMRKASAK
Medicago CHS2                        NSIFWIAHPGGPAILDQVEQKLALKPEKMNATREVLSEYGNMSSACVLFILDEMRKKSTQ
Conium PKS2                          NSIFWIAHPGGPTILDQIESELSLKAEKLKCTRQVLRDFGNLSSACVLFILDEMRKASAK
Gerbera CHS1                         NSIFWIAHPGGPAILDQVELKLGLKEEKLRATRHVLSEYGNMSSACVLFIIDEMRKKSSE
Conium PKS1                          NSLFWIAHPGGPAILDQVESKLGLKEEKMRATRQVLSDYGNMSSACVLFILDEMRKKSIE
Gerbera 2PS                          NSVFWMVHPGGRAILDQVERKLNLKEDKLRASRHVLSEYGNLISACVLFIIDEVRKRSMA
Spsittacina-10591_isotig10557        ------------------------------------------------------------
```

```
Spsittacina-26038_isotig10557         ------------------------------------------------------------
Spurpurea-22109_isotig09797           ------------------------------------------------------------
Spurpurea-9803_isotig09797            ------------------------------------------------------------
Spurpurea-475_isotig00474             NSLFWIAHPGGPAILDKVEEKLALKPEKLRATRHVLSEYGNMSSACVLFILDEMRKKSAE
Spurpurea-12781_isotig00474           NSLFWIAHPGGPAILDKVEEKLALKPEKLRATRHVLSEYGNMSSACVLFILDEMRKKSAE
Spurpurea-478_isotig00477             NSLFWIAHPGGPAILDKVEEKLALKPEKLRATRHVLSEYGNMSSACVLFILDEMRKKSAE
Spurpurea-12783_isotig00476           NSLFWIAHPGGPAILDKVEEKLALKPEKLRATRHVLSEYGNMSSACVLFILDEMRKKSAE
Spurpurea-477_isotig00476             NSLFWIAHPGGPAILDKVEEKLALKPEKLRATRHVLSEYGNMSSACVLFILDEMRKKSAE
Spurpurea-476_isotig00475             NSLFWIAHPGGPAILDKVEEKLALKPEKLRATRHVLSEYGNMSSACVLFILDEMRKKSAE
Spurpurea-12782_isotig00475           NSLFWIAHPGGPAILDKVEEKLALKPEKLRATRHVLSEYGNMSSACVLFILDEMRKKSAE
Spurpurea-12784_isotig00477           NSLFWIAHPGGPAILDKVEEKLALKPEKLRATRHVLSEYGNMSSACVLFILDEMRKKSAE
Spsittacina-5295_isotig05263          NSLFWIAHPGGPAILDKVEEKLALKPEKLRATRHVLSEYGNMSSACVLFILDEMRKKSAE
Spsittacina-5294_isotig05262          NSLFWIAHPGGPAILDKVEEKLALKPEKLRATRHVLSEYGNMSSACVLFILDEMRKKSAE
Spsittacina-20742_isotig05263         NSLFWIAHPGGPAILDKVEEKLALKPEKLRATRHVLSEYGNMSSACVLFILDEMRKKSAE
Spsittacina-20741_isotig05262         NSLFWIAHPGGPAILDKVEEKLALKPEKLRATRHVLSEYGNMSSACVLFILDEMRKKSAE
Spurpurea-4182_isotig04177            NSLFWIAHPGGPAILDQVELKLGLKPEKLRATRHVLSEYGNMSSACVLFILDEMRKKSVD
Spurpurea-16488_isotig04177           NSLFWIAHPGGPAILDQVELKLGLKPEKLRATRHVLSEYGNMSSACVLFILDEMRKKSVD
Spsittacina-24513_isotig09032         NSLFWIAHPGGPAILDQVELKLGLKPEKLRATRHVLSEYGNMSSACVLFILDEMRKKSVE
Spsittacina-9066_isotig09032          NSLFWIAHPGGPAILDQVELKLGLKPEKLRATRHVLSEYGNMSSACVLFILDEMRKKSVE

                                      361      370       380       390       400       410       420
                                      |        |         |         |         |         |         |
Conium PKS5                           DGKPTTGEGLDWGVLFGFGPGLTVETVVLHSVPT--------------------------
Medicago CHS2                         NGLKTTGEGLEWGVLFGFGPGLTIETVVLRSVAI--------------------------
Conium PKS2                           DGKRSTGEGLDWGVLFGFGPGLTVETVVLHSVPT--------------------------
Gerbera CHS1                          NGAGTTGEGLEWGVLFGFGPGLTVETVVLHSVPTTVTVAV--------------------
Conium PKS1                           EGKATTGEGLDWGVLFGFGPGLTVETVVLHSVPATITQ----------------------
Gerbera 2PS                           EGKSTTGEGLDCGVLFGFGPGMTVETVVLRSVRVTAAVANGN------------------
Spsittacina-10591_isotig10557         ------------------------------------------------------------
Spsittacina-26038_isotig10557         ------------------------------------------------------------
Spurpurea-22109_isotig09797           ------------------------------------------------------------
Spurpurea-9803_isotig09797            ------------------------------------------------------------
Spurpurea-475_isotig00474             NGLKTTGEGLEWGVLFGFGPGSPSRLLCSTA-----------------------------
Spurpurea-12781_isotig00474           NGLKTTGEGLEWGVLFGFGPGSPSRLLCSTA-----------------------------
Spurpurea-478_isotig00477             NGLKTTGEGLEWGVLFGFGPGSPSRLLCSTA-----------------------------
Spurpurea-12783_isotig00476           NGLKTTGEGLEWGVLFGFGPGSPSRLLCSTA-----------------------------
Spurpurea-477_isotig00476             NGLKTTGEGLEWGVLFGFGPGSPSRLLCSTA-----------------------------
Spurpurea-476_isotig00475             NGLKTTGEGLEWGVLFGFGPGSPSRLLCSTA-----------------------------
Spurpurea-12782_isotig00475           NGLKTTGEGLEWGVLFGFGPGSPSRLLCSTA-----------------------------
Spurpurea-12784_isotig00477           NGLKTTGEGLEWGVLFGFGPGSPSRLLCSTA-----------------------------
Spsittacina-5295_isotig05263          NGLKTTGEGLEWGVLFGFGPGLTVETVVLHSLST--------------------------
Spsittacina-5294_isotig05262          NGLKTTGEGLEWGVLFGFGPGLTVETVVLHSLST--------------------------
Spsittacina-20742_isotig05263         NGLKTTGEGLEWGVLFGFGPGLTVETVVLHSLST--------------------------
Spsittacina-20741_isotig05262         NGLKTTGEGLEWGVLFGFGPGLTVETVVLHSLST--------------------------
Spurpurea-4182_isotig04177            DGLKTTGEGLEWGVLFGFGPGLTVETVVLHSVTA--------------------------
Spurpurea-16488_isotig04177           DGLKTTGEGLEWGVLFGFGPGLTVETVVLHSVTA--------------------------
Spsittacina-24513_isotig09032         DGLKTTGEGLEWGVLFGFGPGLTVETVVLHSVTA--------------------------
Spsittacina-9066_isotig09032          DGLKTTGEGLEWGVLFGFGPGLTVETVVLHSVTA--------------------------
```

Fig S3. Alignment of *Sarracenia* PKSs with selected plant-PKSs translated into an amino acid sequence. Conserved amino acids of the active site are bolded and coloured amino acids indicate mutated amino acids of the active site. GenBank accession numbers: *Conium maculatum* CPKS1 (KP726914), *Conium maculatum* CPKS2 (KP726915), *Conium maculatum* CPKS5 (KP726916), *Gerbera hybrida* 2PS (CAA86219.2), *Gerbera hybrida* CHS1 (Z38096.1), *Medicago sativa* CHS2 (L02902.1).
